# Terrestrial connectivity, upstream aquatic history and seasonality shape bacterial community assembly within a large boreal aquatic network

**DOI:** 10.1038/s41396-021-01146-y

**Published:** 2021-11-02

**Authors:** Masumi Stadler, Paul A. del Giorgio

**Affiliations:** Groupe de Recherche Interuniversitaire en Limnologie (GRIL), Département des Sciences Biologiques, Université du Québec à Montréal, Montréal, QC Canada

**Keywords:** Limnology, Microbial ecology, Freshwater ecology

## Abstract

During transit from soils to the ocean, microbial communities are modified and re-assembled, generating complex patterns of ecological succession. The potential effect of upstream assembly on downstream microbial community composition is seldom considered within aquatic networks. Here, we reconstructed the microbial succession along a land-freshwater-estuary continuum within La Romaine river watershed in Northeastern Canada. We captured hydrological seasonality and differentiated the total and reactive community by sequencing both 16 S rRNA genes and transcripts. By examining how DNA- and RNA-based assemblages diverge and converge along the continuum, we inferred temporal shifts in the relative importance of assembly processes, with mass effects dominant in spring, and species selection becoming stronger in summer. The location of strongest selection within the network differed between seasons, suggesting that selection hotspots shift depending on hydrological conditions. The unreactive fraction (no/minor RNA contribution) was composed of taxa with diverse potential origins along the whole aquatic network, while the majority of the reactive pool (major RNA contribution) could be traced to soil/soilwater-derived taxa, which were distributed along the entire rank-abundance curve. Overall, our findings highlight the importance of considering upstream history, hydrological seasonality and the reactive microbial fraction to fully understand microbial community assembly on a network scale.

## Introduction

Microbial communities across ecosystems are characterized by rank abundance distributions that vary in shape, yet we still know relatively little about how these structures come to be. Distribution shapes are thought to provide insight on community assembly [1], with dominant and rare taxa assumed to be locally successful and transient, respectively [2, 3], but these interpretations have seldom been explicitly confirmed. Inherited from macroecology, microbial assembly processes have been defined into four fundamental categories—selection, dispersal, diversification, and drift—[4], which vary in their degree of determinism and stochasticity [5]. Whereas diversification and drift usually manifest on longer, evolutionary, time scales, selection as well as dispersal are more relevant on ecological time scales. Regardless, there is always a historical aspect to community assembly as local microbial communities reflect the balance between selection and dispersal processes that have occurred locally and in connected habitats in the past [6]. Hence, accounting for community history is vital to understand community assembly and the shape of the rank abundance distribution.

Studies that investigated the relevance of history mainly followed microbes across time within an ecosystem [7, 8]. Temporal history does shape local communities (i.e., legacy effects [9]), however, within an aquatic network, the uni-directional flow of water links temporal and spatial histories. Hydrology is a major driver of aquatic microbial community composition [10, 11], as evidenced by soil microbes being flushed into and representing large proportions of aquatic communities [12–15]. As such, community structure at any given site within a hydrological network is the net result of upstream assembly processes [16], and network connectivity is further modulated by seasonal hydrological fluctuations [17, 18]. Therefore, spatial history is particularly relevant in highly interconnected freshwater networks [19], and there have been various studies that investigated the spatial context of aquatic microbial community assembly. Stegen et al. [20] quantified major assembly processes based on spatial patterns of phylogenetic as well as taxonomic dispersion, which assumes that phylogenetically related organisms have similar niche requirements. Others have used spatial numerical distributions to infer the relative importance of selection versus passive transport across separate watersheds [21]. While the importance of mixing and interacting communities between different ecosystems is now amply recognized (i.e., community coalescence [22]), few studies consider interfaces between multiple ecosystems or ecosystem domains (e.g., terrestrial—aquatic) [23, 24]. A spatially connected, true aquatic continuum has mostly been evaluated on local scales within lakes [25–27], along a single river mainstem [28–32] or on interconnected upstream networks [14, 16, 33–36] and rarely have surrounding terrestrial ecosystems been considered as potential sources [12, 13, 15]. Moreover, active and passive assembly processes are difficult to resolve as cell death and dormancy blur interpretations based on DNA patterns alone [37, 38]. Indicative of recent protein synthesis, RNA sequencing has helped to disentangle active from unreactive microbial members [39], however, only few freshwater studies have included both [15, 25, 40–44]. All of these studies have separately yielded useful insight on microbial community assembly in freshwater systems, and they collectively point to the challenges ahead.

The processes shaping community assembly are dynamic; selection and mass effects will vary in relative importance along complex aquatic networks as a function of the degree of connectivity to surrounding ecosystems, and upstream history. In order to capture the shifting balance of assembly processes and link those to the underlying rank abundance structure, we first need to examine a true hydrologic continuum that includes source communities and exchanges between various aquatic as well as terrestrial habitats as potential sources. Secondly, seasonality needs to be accounted for as the degree of connectivity depends largely on various hydrological scenarios in these networks. And lastly, DNA has to be accompanied by some indication of reactivity as selection and passive dispersal cannot be fully distinguished otherwise. In this study, we attempted a more holistic approach to aquatic microbial community assembly by addressing the three aforementioned critical dimensions.

### Conceptual framework

Our overall aim was to follow shifts in bacterial community structure along a terrestrial-aquatic continuum and assess how the relative importance of mass effects versus species selection changes as communities traverse through varying environmental conditions and degrees of connectivity to the surrounding catchment. We carried out this study within La Romaine river watershed in the Northeastern region of boreal Québec, Canada, over several years and seasons. Starting from upstream sources such as soils, soilwaters, and headwater streams, we continued to follow the extant river orders (Strahler order 0–7) up to the estuarine plume. In addition, three reservoirs have been consecutively flooded mid-river over the sampling period. The sampling design covers various interfaces (terrestrial-aquatic, stream-river, river-reservoir, freshwater-estuary), and other ecosystems within the watershed (e.g., headwater ponds, tributaries, lakes) that provide a further meta-community context.

We first assess how the 16 S rRNA gene (DNA-based) assemblage structure shifts along the terrestrial-freshwater-estuary continuum; we determine the general patterns of the spatial succession and its relation to different hydrologic seasonality. Furthermore, we differentiate the reactive from the total bacterial assemblage by additionally examining the 16 S ribosomal RNA patterns relative to DNA. In this regard, RNA is not being used as an indication of absolute activity [45], rather we interpret the patterns of convergence and divergence between assemblage structures based on DNA and RNA (hereafter, DNA-RNA-based assemblage structures) along the continuum to infer shifts in the relative importance of species selection versus mass effects. To quantify divergence between DNA-RNA-based assemblage structures, we computed the distance between each DNA and RNA pair within multivariate space based on either incidence (presence-absence) or abundance dissimilarities. In a null scenario where DNA and RNA follow the exact same patterns, DNA-RNA-based assemblage structures remain equidistant, which would indicate no influx of unreactive bacteria (i.e., only detectable in DNA), and no changes in the reactivity of taxa within the community (no inactivation and activation of active and dormant taxa, respectively). Local divergence in DNA-RNA-based assemblage structures in the continuum, on the other hand, may result from an influx of bacteria unreactive to local conditions (low RNA detectability), which would strongly influence the incidence-based distance, or from local shifts in the reactivity of specific taxa within the community (local activation/inactivation of taxa), influencing mostly the abundance-based distance. Shifts in how the incidence- and abundance-based metrics relate to each other across space and time, enabled us to gain insight into when and where selection or mass effects outweigh the other along the continuum. Finally, we explore where taxa potentially originated along the continuum and what fraction within the rank abundance curve the unreactive and reactive taxa commonly occupy.

## Material and methods

### Catchment characteristics and sampling

**Fig. 1:**
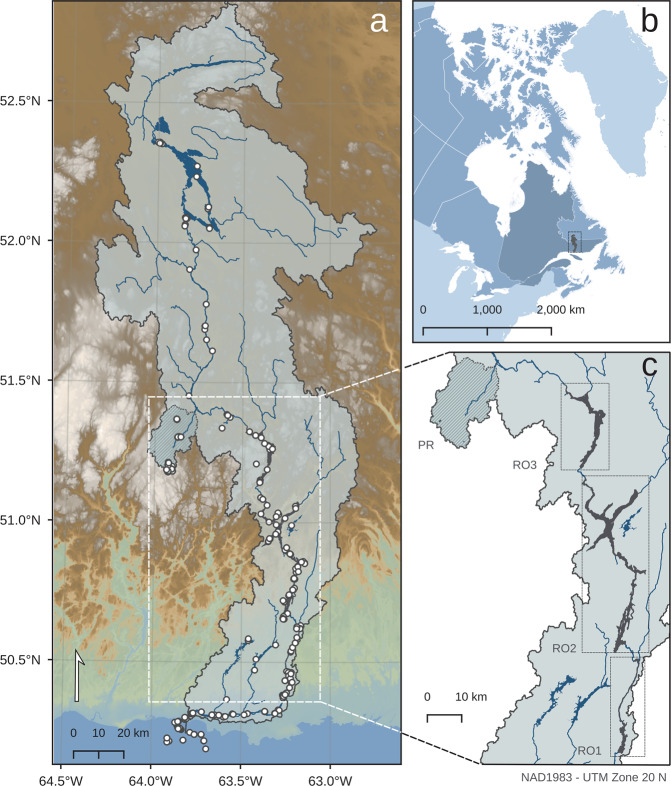
Location and overview of the La Romaine catchment. **a** Scale and overview of the whole La Romaine catchment. Samples are represented as points. **b** Location of the catchment within Canada and Québec. **c** Focus on all built reservoirs RO1 (2015), RO2 (2014) and RO3 (2017) and the headwater stream sub-catchment Petite Romaine (PR).

To follow the movement of microbial communities within a watershed, samples were taken along La Romaine river (Strahler order 7, Côte-Nord region, Québec, Canada) (Fig. 1a, b) from 2015-2017. La Romaine catchment belongs to the eastern black spruce-moss bioclimatic domain and has an area of ~14,500 km^2^. For detailed catchment characteristics refer to the supplementary methods (hereafter, SM) (SM1, Fig. S1). In brief, the mainstem of the river (main trunk of riverine network) flows through a series of large, shallow lakes (hereafter, riverine lakes), emerging as Strahler order 6, and is subsequently dammed in a series of three hydroelectric reservoirs that were consecutively built in 2015 (RO2), 2016 (RO1), and 2017 (RO3). We refer to the river sections before and after the reservoir complex as upriver and downriver, respectively. The river has a total distance from the northern headwaters to the river mouth of ~475 km.

In order to follow a terrestrial-aquatic continuum, various habitat types were sampled (Table S1). To capture a headwater network with soils, soilwaters, streams and ponds, we sampled the Petite Romaine sub-catchment (PR, *A*: 310.73 km^2^, elevation: 580 masl, Strahler orders 0–4, Fig. 1c) due to the remoteness and inaccessibility of the northernmost headwaters. By sampling this headwater sub-catchment, we were able to follow a true continuum from an example headwater stream to the mainstem river into the estuarine plume. In addition, we sampled the reservoirs that are located along the mainstem. Other sites such as groundwaters, tributaries (Strahler orders 1–5), lakes and sediments in the catchment were sampled for a meta-community context. Tributaries refer to streams and rivers that were sampled at the confluence of the mainstem, in contrast to streams within the PR sub-catchment that represent a headwater network. Overall, 389 samples were collected for DNA (D) and 201 for RNA (R), covering spring (156-D, 66-R), summer (199-D, 101-R) and autumn (34-D,34-R) (Table S1). RNA samples were collected from 2016 onwards.

For detailed sampling procedures and sample preparation for each sample type refer to SM2. In brief, all water samples were filtered onto a 0.22 µm polycarbonate filter with a peristaltic pump and homogenized soil and sediments were stored in aliquots. RNA samples were submerged in RNAlater and Lifeguard Soil Preservation Solution (Qiagen, Hilden, Germany) for water and non-water samples, respectively. RNA samples were stored at 4 °C overnight to allow stabilization and were subsequently frozen. All DNA and RNA samples were frozen at −20 °C at the field station and further stored at −80 °C at the university laboratory until extraction. DNeasy and RNeasy PowerWater and PowerSoil kits (QIAGEN, Hilden, Germany) were used following the manufacturer’s instructions. RNA extracts were reversely transcribed to cDNA with a high capacity cDNA Reverse Transcription Kit (Applied Biosystems, Foster City, CA, USA) and all samples were sent to Génome Québec Innovation Center (Montréal, QC, Canada) for paired-end sequencing of the 16 S rRNA V4 region using the primers 515 F (5ʹ-GTGCCAGCMGCCGCGGTAA-3ʹ) and 806 R (5ʹGGACTACHVGGGTWTCTAAT-3ʹ) on a MiSeq platform (PE250, Illumina, San Diego, CA, USA; details in SM2).

### Bioinformatic analysis

A detailed description of the bioinformatic treatment can be found in SM3. In brief, primers were removed from 16 S rRNA DNA and cDNA (hereafter, RNA) reads using *cutadapt* (Version 1.18 [46]). To identify amplicon sequence variants (ASVs), 16 S rRNA amplicon reads were analyzed through the DADA2 (Divisive Amplicon Denoising Algorithm 2) pipeline (Version 1.14.1 [47]). Taxonomy was assigned with the *DECIPHER* package (Version 2.14.0 [48]) implementing the IDTAXA algorithm [49] and the GTDB database (Release 95 [50]). To account for slight differences that may have emerged between DNA and RNA ASVs and potential differences among 16 S rRNA copies within a single genome, ASVs were merged into OTUs by a 99% similarity threshold [51] with the *DECIPHER* package [48]. OTUs only found in RNA (“phantom” taxa) were corrected for by replacing all observations with RNA > 0 and DNA = 0 with DNA = 1 [39].

An observation (i.e., read count of an OTU within a sample) that only appeared in a single sample within each habitat type, season and nucleic acid type combination (i.e., singleton within a factorial combination) was considered unreliable if the singleton OTU had less than 10 reads within the sample. This approach not only removes singletons across the whole database but also singletons within each sampling campaign that had too few reads to be considered reliable. Furthermore, *metagenomeSeq* was used to transform and stabilize variation in library sizes with cumulative sum scaling (CSS) [52] (hereafter: CSS reads). CSS results were compared with results achieved with various rarefaction thresholds and no substantial differences were observed (Fig. S2). A few minor differences are discussed in SM4.

### Data exploration and statistical analyses

A detailed version of this section is included in SM5. To explore differences in microbial community composition across habitat types and seasons, a Principal Coordinates Analysis (PCoA) was conducted with Bray-Curtis dissimilarities (*D*_*BC*_) [53, 54] based on all DNA samples with the function *pcoa* in the *ape* package [55] (*n* = 389, 16,322 OTUs). To evaluate statistical differences in habitat type and season a PERMANOVA was computed with 9999 permutations with the *adonis* function. Multivariate homogeneity was tested for with *betadisper* and *permutest* (*vegan* package [56]). For all statistical analyses, an *α* level of 0.05 was chosen prior to analysis.

To further evaluate whether sampled RNA-based assemblages were different from the DNA-based assemblages, we performed a second PCoA with both DNA and RNA samples (with *D*_*BC*_, *n* = 590, 16,322 OTUs). Again, statistically different groups were investigated with a PERMANOVA (9999 permutations), where habitat type, season and nucleic acid type (DNA vs. RNA) formed the groups. To quantify how different DNA-RNA-based assemblages of the same sample are, the Bray-Curtis distance (m_BC_) of each DNA-RNA sample pair within the PCoA ordination space was computed across *n*-dimensional space [57]; a similar approach to other studies that extracted the magnitude of change in multivariate space between two samples of interest [43, 58]:

$${{{{{{{\mathrm{m}}}}}}}}({{{{{{{\mathrm{p}}}}}}}},{{{{{{{\mathrm{q}}}}}}}}) = \sqrt {(|p_1 - q_1|)^2 + (|p_2 - q_2|)^2 + \cdots + (|p_n - q_n|)^2}$$

where *p* and *q* represent DNA and RNA site scores, respectively, of each sample and *n* is the used maximum number of dimensions. We focused on the first axes that cumulatively explain 75% of the variation for each ordination (n_75%_), similar to Osterholz et al. [59]. This approach was implemented as it was evident from the PCoA that essential variation within non-aquatic samples was captured outside the first three axes and to exclude noise that may be captured when using all dimensions (Fig. S3).

To gain further insight into the processes shaping assemblage dissimilarities, we computed a PCoA with the Sørensen dissimilarity (*D*_*S*_), which is the incidence-based equivalent of *D*_*BC*_ [54, 60] (Fig. S4). By comparing incidence- and relative abundance-based dissimilarities, we can further distinguish in which samples DNA-RNA-based assemblages diverge primarily due to different present taxa or their abundances, respectively. We further applied the same framework of calculating the distance among DNA and RNA pairs across n_75%_ axes resulting in the Sørensen-based distance (m_S_) (Fig. S4). In order to examine where shifts in the relative importance between incidence- and relative abundance-based distances were happening along the continuum, we calculated the difference between m_BC_ and m_S_ (∆-distance). To explore the interpretability of the ∆-distance approach, we simulated theoretical communities and computed ∆-distances for this mock dataset (details in SM6). In brief, four species abundance distributions (SADs) with various levels of evenness were created and randomly sampled to generate DNA assemblages. We hypothesized that these various SADs represent a gradient from mass effects to selection, where less even communities are linked to stronger selection. Subsequently, DNA assemblages were duplicated for each site to create a base for the corresponding RNA assemblage. We hypothesized that the higher the number of OTUs in DNA without RNA, the stronger the mass effect. To create an additional range of mass effects, different numbers of OTUs were removed from the RNA assemblage. Results indicated that lower ∆-distance values correspond to stronger mass effects as indicated by higher replacement and higher evenness. Inversely, higher ∆-distance values indicate stronger selection with lower replacement and lower evenness (Figs. S5–S6). Results obtained during our rarefaction test (SM4) showed that absolute numbers of ∆-distances varied across rarefaction thresholds, while relative patterns across seasons and habitat types remained consistent. Hence, absolute values in ∆-distances are likely to hold little meaning, and it is rather the relative change in ∆-distances and the resulting pattern across gradients or between habitats that is informative and comparable across studies.

### Abundance groups

In order to explore where within a rank abundance curve community reshuffling is occurring, abundance groups (e.g., abundant, moderate, rare) were defined based on the shape of rank abundance curves per habitat type. Abundance thresholds are defined as the first and second moment of maximum acceleration along the rank abundance curve (Fig. S7). This approach classified all OTUs with > = 72 CSS reads as abundant, <72 and > = 10 CSS reads as moderate, and <10 CSS reads as rare (details in SM7). This classification method was implemented as abundance thresholds commonly used are rather inconsistent across the literature, with little confidence in whether a particular abundance threshold is suitable for a given dataset. The implemented approach is not different from common fixed relative abundance thresholds (e.g., 1%), the only difference lies in the fact that the abundance threshold is derived empirically from the species abundance distributions of the studied dataset.

### Classification of OTU origin and potential reactivity

OTUs were classified by the habitat in which they were first detected along the terrestrial-aquatic continuum (regardless of season) to have a proxy of origin for each OTU (hereafter, potential origin) [12, 13]. The classification followed the order of soil, soilwater, stream, upriver, reservoirs, downriver, and finally, the estuary.

To further explore patterns in the OTUs’ DNA and RNA relationship, we correlated the contribution of individual OTUs to each local DNA and RNA pool (e.g., a local pool was defined for each season and habitat). The OTU contributions to each local pool were first averaged for each potential origin, abundance group and four RNA contribution categories (<25% confidence interval (CI), < median, > median and >75% CI) to enhance visibility. On first attempt, all OTUs falling in the <25% CI and < median categories showed striking invariability, while >75% CI and > median categories followed a linear relationship between an OTU’s contribution to the DNA and RNA pools. This pattern was observed across most local pools, and hence, the contribution categories were reduced to two groups (>median and <median). This categorization threshold is referred to as the ‘potential reactivity threshold’ (hereafter PRT, median: 0.067%), based on the apparent decoupling of DNA and RNA of OTUs below the median. We infer that the absence of a relationship between DNA and RNA likely reflects that these taxa below the PRT are present but may be generally unreactive to the environment, given that their numerical abundance is largely unrelated to their apparent potential activity. Hence, we categorized the taxa below the PRT together with taxa that did not have any RNA as “unreactive”. It is important to note here that the PRT was applied for each habitat and season, hence, an OTU classified as reactive within for example soils, may become unreactive or stay reactive in subsequent habitats or seasons.

All analyses have been conducted in R v3.4.2 [61] and RStudio v1.3.1073 [62] (package details in SM8).

## Results

Sampled sites covered a large range of habitat types from soils, soilwater, over streams, the main river, lakes, reservoirs and the estuary. We recovered 51,901,843 quality filtered reads, with 119,109 identified ASVs. After 99% similarity OTU clustering, there were 35,995 unclassified OTUs that were removed in the downstream analyses. After sub-sampling only bacteria, 48,927,604 reads and 16,322 OTUs were retained. The smallest and largest library size were found in a sample of riverine lakes and soilwater with 1470 and 81,716 reads, respectively. On average, the lowest library sizes were found in sediment, soil, soilwater and estuary with less than 20,000 reads. In contrast, most freshwater samples had a library size larger than 20,000 reads (Fig. S8). There were 56 phyla, 130 classes, 316 orders, 571 families, and 1027 genera represented in the dataset. Relative abundances of phyla varied across habitat types (Fig. S8) but on average, the meta-community across all ecosystems was composed of Proteobacteria (38.4%), Verrucomicrobiota (9.6%), Patescibacteria (7.9%), Acidobacteriota (5.3%), Myxococcota (5.2%), Actinobacteriota (5.2%), Bacteroidota (4.5%), Bdellovibrionota (3.8%), Planctomycetota (3.2%) and Cyanobacteria (2.7%).

### Gradual change of assemblage structures along the terrestrial-aquatic continuum

**Fig. 2:**
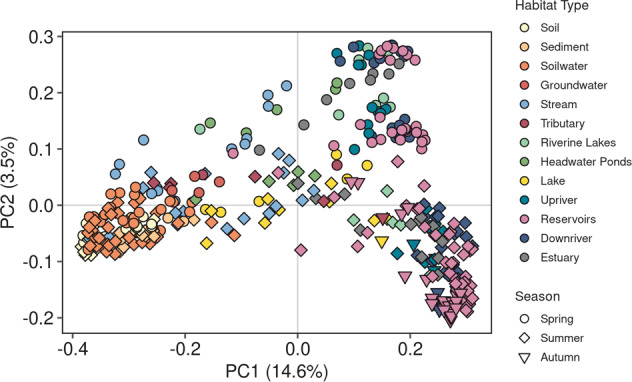
Microbial community composition gradually changes along a terrestrial-hydrological continuum and diverges between seasons. Overall PCoA analysis of DNA samples. The PCoA reveals microbial community shifts from terrestrial to freshwater samples. Habitat types ranged from soils, soilwaters, streams and headwater ponds (sampled in headwater stream network, Stahler orders 0-4), tributaries (sampled at the confluence to the mainstem, Strahler orders 1–5), riverine lakes (northernmost lakes through which the Romaine river flows), reservoirs, sections along the mainstem (Strahler order 6–7) upstream and downstream of the reservoir complex (upriver and downriver, respectively), and the estuary. Spring and summer/autumn show distinct paths in multivariate space. Percentage of variance explained are given in brackets for the first and second axes.

We observed a clear directional pattern in community composition based on DNA, from the most terrestrially-influenced habitats such as soil, soilwater and sediment to the mainstem river and reservoir sites and the estuary, which was captured in the first PCoA axis (Fig. 2) and statistically supported by a PERMANOVA analysis (Table 1). Groundwaters, streams, tributaries, headwater ponds, and lakes were clearly aligned between the two endpoint clusters formed by terrestrial and riverine/reservoir samples (Fig. 2).

**Table 1:** PERMANOVA and PERMDISP results based on DNA alone and DNA and RNA community matrices.

Dataset	Group	PERMANOVA				PERMDISP		
		*df*	*F-*statistic	*R*^*2*^	*p* value	*df*	*F-*statistic	*p* value
DNA	Habitat	12	17.5	0.35	<0.0001	12	38.9	<0.0001
	Season	2	12.1	0.04	<0.0001	2	58.4	<0.0001
	Combined					27	20.7	<0.0001
DNA and RNA	Habitat	12	20.0	0.28	<0.0001	12	28.9	<0.0001
	Season	2	15.6	0.04	<0.0001	2	62.0	<0.0001
	Nucleic acid type	1	25.6	0.03	<0.0001	1	1.2	0.27
	Combined					49	9.6	<0.0001

The directional trajectory along the terrestrial-aquatic continuum had a striking seasonality that was especially marked for the downstream river and reservoir sites, with a clear separation between spring and summer/autumn samples along the second PCoA axis (Table 1). Soil, sediment, soilwater and groundwater sites, however, did not exhibit a clear seasonality (Fig. 2) and seasonality did not emerge as a strong driver even within a PCoA performed only with terrestrially-influenced samples (Fig. S9). Although PERMANOVA results strongly supported habitat type and seasonal clustering, the results could be affected by different dispersion of data within multivariate space, which interferes with a straight forward interpretation of the results as a PERMANOVA cannot distinguish among-group from within-group variation if data dispersion is variable [63]. Differences in dispersion were found by habitat type alone, solely season and both habitat and season combined (Table 1). While dispersion between spring and summer was not statistically different (Tukey Honestly Significant Difference: *p* > 0.05), dispersion was always different when comparing autumn with other seasons (Tukey Honestly Significant Difference: *p* < 0.0001). The average distance of samples within the autumn cluster to its median was smaller compared to other seasons (0.46 vs 0.63/0.62) likely due to a smaller sample size in autumn (*n*_autumn_ = 34 vs. *n*_spring_ = 156, *n*_summer_ = 199). Among habitat types, dispersion was larger in terrestrially-influenced sites such as soil (Distance to median: 0.61), soilwater (0.63), stream (0.63) and tributary (0.62) samples, compared to riverine (0.51), reservoir (0.52) and estuary (0.52) samples. The observed differences in the heterogeneity within habitat types likely reflects inherent characteristics of these ecosystems, with sites of stronger terrestrial influence exhibiting stronger spatial variance.

### Patterns in RNA and DNA divergence

**Fig. 3:**
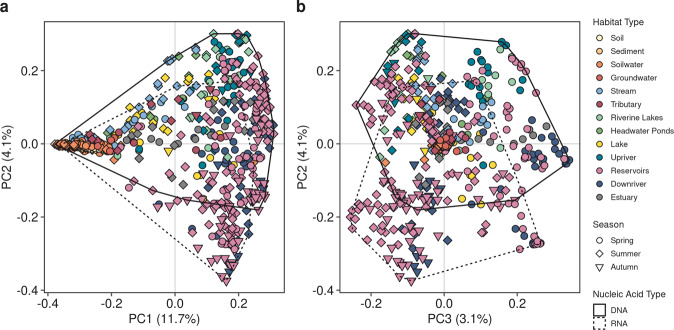
RNA assemblages diverge from DNA within aquatic habitats, less so in terrestrially-influenced habitats. PCoA analysis including RNA samples. **a** Visualization of first and second axes of PCoA, differentiating habitat type and nucleic acid type, respectively. **b** Different view on PCoA analysis using the second and third axes, differentiating nucleic acid type and seasons, respectively. The distribution of DNA and RNA samples are highlighted with polygons. Percentage of variance explained by the corresponding axes are given in brackets.

When DNA and RNA samples were combined in a second PCoA analysis, the three main axes of variation were habitat type (PC1), nucleic acid type (PC2) and seasons (PC3), and these three first axes captured in summary 18.9% of the dissimilarity variance (Fig. 3). Strong DNA-RNA divergence emerged as soon as the continuum enters the upstream aquatic sites (i.e., streams) and amplified along the continuum (Fig. 3a). Seasonality was most pronounced in aquatic sites in both DNA and RNA (Fig. 3b) and was the second-strongest driver after the spatial continuum in a PCoA only with RNA samples (Fig. S10). There was no clear seasonality and differentiation between nucleic acid types in terrestrially-influenced samples (Figs. 3b, S10). However, it is noteworthy that visual inspection indicated that much of the terrestrially-influenced site dissimilarity was split upon additional axes (data not shown). Overall, PERMANOVA analysis indicated significant clustering by habitat type, season and nucleic acid type (Table 1). Similar to the DNA only PCoA, homogeneity of dispersion was mostly not fulfilled [63]. According to PERMDISP, dispersion differed by all factorial combinations, habitat type and season, however, not for nucleic acid type alone (Table 1). Dispersion patterns among seasons and habitats were similar to the DNA only PERMDISP results, where dispersion was smaller in autumn compared to spring and summer and terrestrially influenced sites generally had a larger dispersion.

### Inferring assembly dynamics from DNA-RNA pair-wise distance patterns along the continuum

**Fig. 4:**
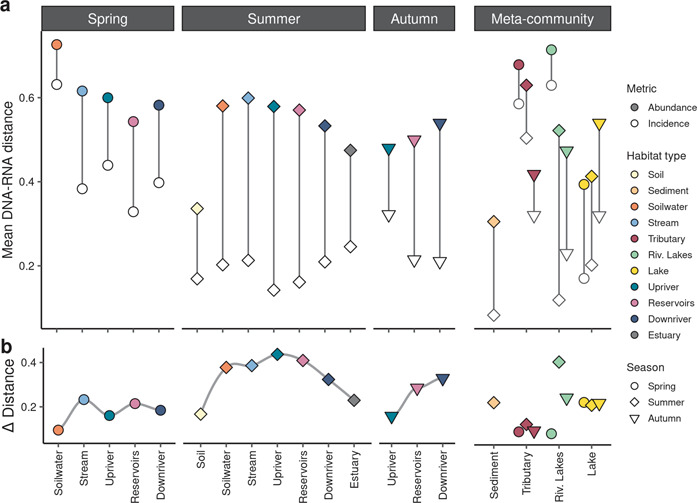
Patterns between abundance and incidence-based distance reveal seasonal shifts in assembly processes. **a** Distances between DNA and RNA of the same sample within ordination space were averaged by habitat type and season. Abundance-based (colored points, m_BC_) and incidence-based distance (hollow points, m_S_) indicate spatio-seasonal trends in strong mass effects, especially in spring with high incidence-based distances. **b** ∆-distances between m_BC_ and m_S_ indicate shifts in the relative contribution of incidence- vs. abundance-based distances along the continuum, with high ∆-distances indicating stronger selective forces. Habitats sampled outside the direct continuum are given as an additional meta-community context.

To further explore the patterns in DNA-RNA-based assemblage structure differences along the continuum, we calculated the distance in PCoA ordination space between DNA and RNA of each sample based on abundance (m_BC_) and incidence (m_S_) along the number of axes capturing 75% of variance (n_75%_) as the depicted first three axes in Fig. 3 only captured a limited fraction of the total DNA-RNA divergence (n_75%_) axes: Sørensen = 204, Bray-Curtis = 192. Based on the incidence metric m_S_, the largest average distances between DNA and RNA were found in spring (0.43 ± 0.29 (mean ± standard deviation)) especially in soilwaters (0.63 ± 0.33). In contrast, summer m_S_ distances were in general lower (0.18 ± 0.12) and more similar among habitat types (Fig. 4a). The abundance-based distance (m_BC_) was in general very similar among seasons with spring being slightly higher (0.61 ± 0.20) than summer (0.53 ± 0.15) and autumn (0.50 ± 0.06). Lowest m_BC_ distances were found in soil (0.34) and sediment (0.30) samples. Overall, m_BC_ values were always larger than m_S_, as m_BC_ captures both abundance and incidence differences and hence, add abundance-based differences to the distance observed with m_S_.

To investigate relative changes between incidence and abundance-based distances, we computed the difference between m_BC_ and m_S_ (∆-distance)(Fig. 4b). The ∆-distance can theoretically range between 1 and -1, both exemplifying extreme cases where m_BC_ = 1 and m_S_ = 0 and vice versa. In general, lower (or even negative) ∆-values indicate comparably higher incidence-based distances, where dissimilarity is largely driven by the occurrence of different taxa between DNA and RNA suggesting prevalence of mass effects (SM6). On the other hand, higher ∆-values indicate relatively low incidence-based and high abundance-based distance, representing selection-driven dissimilarity with more taxa common between DNA and RNA but rather large numerical differences (SM6). Hence, we interpret positive shifts in ∆-distances as transition from mass effects to selection dominated habitats and vice versa. Overall, there were different trajectories among seasons in ∆-distances, with lower values in spring remaining relatively stable around 0.17 ± 0.17, and higher average values in summer (0.35 ± 0.16), suggesting relatively higher overall mass effects in spring and selection in summer. In addition, there was a clear spatial pattern in ∆-distance in summer along the continuum, with an increase in ∆-distance from terrestrially-influenced sites (i.e., soils, soilwaters, streams) towards the river followed by a sharp decline downstream of the reservoir, suggesting increasing mass effects downstream of the reservoir. In autumn, selection gradually increases along the mainstem from the upstream river over reservoirs to the downstream river sites. Coinciding with these patterns, other sampled high residence time habitats within the watershed (i.e. riverine lakes, lakes) also had a higher average ∆-distance compared to tributaries (Fig. 4b), suggesting strong selection especially in summer. Whereas lakes remained relatively stable across seasons, riverine lakes had a distinct pattern comparable to tributaries with relatively low ∆-distances in spring indicative of high mass effects.

### Taxa across the rank abundance curve contribute to the reactive pool

**Fig. 5:**
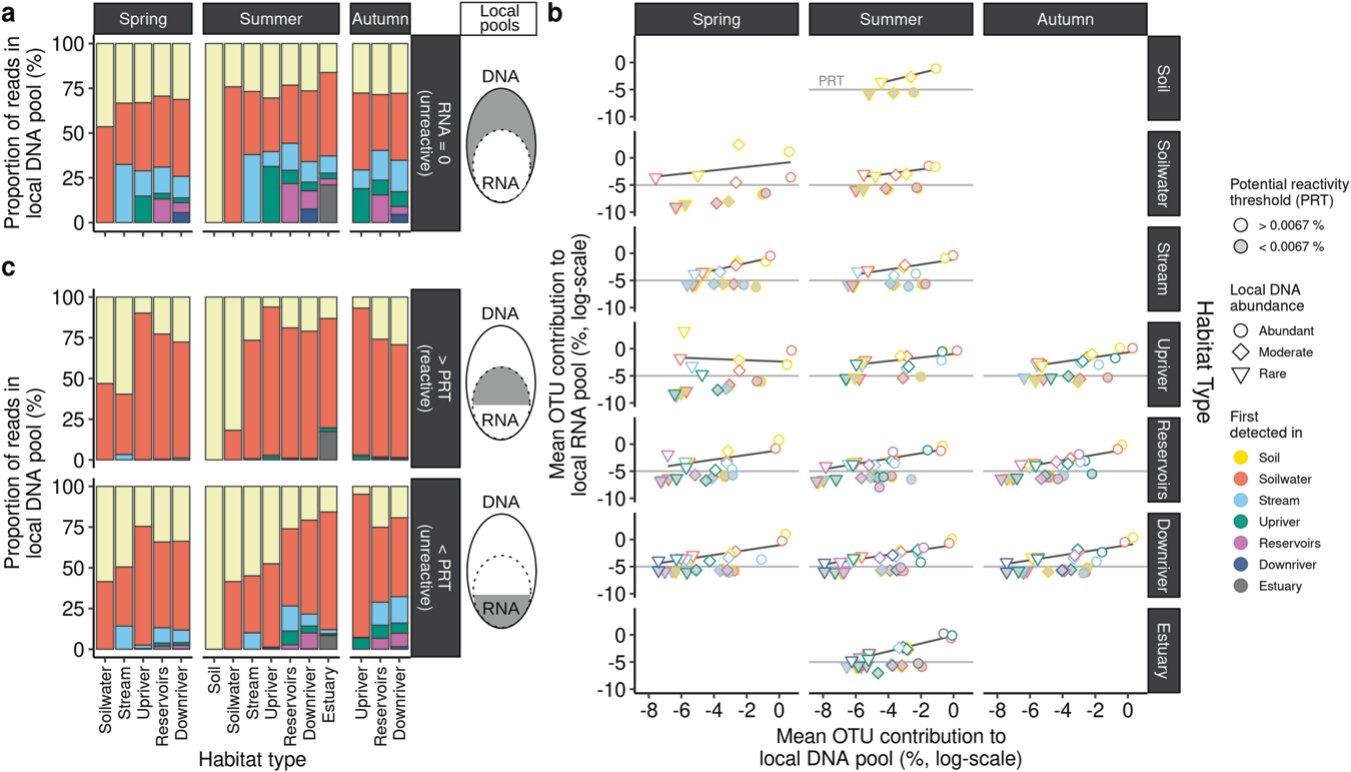
Taxa along the whole rank abundance curve contribute to the reactive pool. **a** Contribution of taxa (operational taxonomic units (OTUs)) detected in DNA without any RNA observation (RNA = 0) to the local number of DNA reads for each habitat type and season. OTUs were binned by (1) their first detected habitat in DNA along the continuum, (2) local DNA abundance (e.g. abundant, moderate, rare, see methods). **b** Each panel corresponds to an individual OTU’s reads contribution to the local DNA and RNA pool, which was defined for each season and habitat type. Taxa were classified and averaged by (1) first detected habitat and (2) local DNA abundance and lastly, (3) by whether they are above or below the identified potential reactivity threshold (PRT) of 0.0067% RNA contribution to the total local RNA pool. Linear regression slopes were plotted for the averaged OTU contributions above the PRT. Due to the absence of a linear relationship of OTUs < PRT, OTUs > PRT were interpreted to be reactive, while OTUs < PRT were considered unreactive. **c** Based on the observations of (**b**) and the consequent classification of reactive (upper panel) and reactive (lower panel) taxa, individual OTU’s contribution to the total local DNA pool were visualized. Values are expressed as a fraction of the total number of DNA reads for each habitat type and season.

In the previous sections we have established that there were patterns of divergence and convergence of DNA-RNA-based assemblage structure that were spatially and temporally structured. To further explore what fractions within the community contribute to mass effects and selection, respectively, we classified OTUs by the habitat in which they were first detected along the terrestrial-aquatic continuum, from soils to the estuary. In particular, by focusing on taxa that were only detected in DNA (termed unreactive) and thus strongly contribute to the observed mass effects, it became evident that a relatively large proportion potentially originated in the upstream habitats such as soils, soilwaters and streams. Yet new unreactive taxa were also gained along the entire continuum (Fig. 5a).

In order to understand the coherence between DNA and RNA patterns of reactive taxa that contribute to the observed patterns in selection, we examined the relative contribution of individual taxa to the DNA and RNA sequence pools for each habitat and season (Fig. 5b). For this, we also grouped OTUs according to where they were first detected in DNA along the continuum to identify subsets of taxa that activated and developed locally. To simplify the analysis, we further classified OTUs based on their mean DNA habitat abundance into locally abundant, moderate and rare taxa (see “Methods”). Inspection of the various plots in Fig. 5b revealed a recurrent pattern in most habitats with a subset of taxa present across all DNA abundance groups, which nevertheless contributed negligibly to the local RNA pool (between 0.00005 and 0.0067% of sequences) and whose RNA contribution was decoupled from their contribution to the DNA pool. In contrast, there was another subset of taxa with a consistently higher contribution to %RNA sequences (above 0.0067% sequences, hereafter termed reactive) and whose contribution to RNA and DNA pools appeared to be linearly coupled. Although we classify these two groups as “reactive” and “unreactive” out of simplicity, we do not know why some taxa have a decoupled DNA and RNA relationship. We acknowledge that taxa within the “unreactive” fraction may be taxa with disproportionately low rRNA albeit being still active. Reactive taxa were represented across DNA abundance groups, and in most habitats the overall relationship between %DNA and %RNA contribution averaged around a log-log slope of 1.18, suggesting a roughly proportional contribution (Fig. 5b), except for upriver spring samples where numerically rare taxa appeared to have disproportionately high RNA. On the other hand, taxa that were unreactive had a striking invariance, hovering around 0.0025% RNA contribution which may indicate a lower activity threshold. These taxa may be somewhat analogous to taxa that were entirely not detected in RNA and only in DNA. Taxa without a single detected RNA copy can have RNA in the environment, however, their RNA content may be too low to be captured with our sequencing depth. The reactive and unreactive taxa were both distributed across the entire range of %DNA contribution. The former indicates that there are locally reactive OTUs across the entire rank abundance curve, including rare taxa. Likewise, there were unreactive taxa across the entire rank abundance curve that may be numerically important in DNA. Furthermore, reactive taxa occupy on average 45.9% of the OTU pool in each habitat, whereas the unreactive taxa (<0.0067% contribution (22.7%) and RNA = 0 (31.4%)) together comprise 54.1% (Fig. S11).

Within the reactive fraction, OTUs that were first detected in soil had the largest overall contributions to the local RNA pool of reactive taxa across all habitats and seasons (overall mean of 0.97%) followed by soilwater-derived OTUs (0.22%), but OTUs that were first detected in a given local habitat were found within the reactive fraction of all DNA abundance groups (Fig. 5b). Soilwater and soil-derived taxa were less prevalent in the estuary, where riverine and estuarine-derived taxa become numerically more important in RNA. The overwhelming majority (>90%) of DNA sequences of reactive taxa along the entire continuum (except for estuarine sites) could be retraced to OTUs that were first detected in soils and soilwaters (Fig. 5c, top panel). In contrast, a relatively large proportion of DNA sequences of unreactive taxa were contributed by a more diverse pool of OTUs that originated in various habitats along the continuum (Fig. 5c, bottom panel), a pattern similar to that of OTUs undetected in RNA (Fig. 5a). The latter would suggest a high level of influx and persistence of OTUs along the continuum that were seemingly unreactive. Interestingly, there was not a single OTU that remained abundant or moderately abundant along the entire continuum, suggesting that the soil/soilwater-derived taxa, which consistently dominate the reactive fraction, nevertheless shift along the rank abundance curve along the continuum.

## Discussion

In this study, we attempted to address three major challenges identified in understanding microbial community assembly within aquatic networks: First, to incorporate the upstream history of local communities, secondly, to capture a variety of hydrological scenarios and thirdly, to capture any indication of reactivity to changing environmental conditions. Here, we followed an interconnected, large scale continuum that extended from upstream soils into the estuary and sampled across seasons to address shifts in assembly processes linked to hydrological fluctuations. In addition, we accompanied DNA with RNA sequencing to distinguish numerical responses that could be linked to passive transport of dormant or inactive bacteria from those associated to reactive taxa [38, 64]. In this regard, we are not using RNA or RNA/DNA ratios as an index of absolute activity [64, 65], but rather utilize spatial patterns in the degree of coupling between DNA-RNA-based assemblages to identify taxa that appear to react to local conditions, and distinguish where selection was most dominant along the network. We further classified OTUs by their habitat in which they were first observed to assess whether taxa from upstream habitats persist along the continuum (i.e., upstream history) and to further examine how unreactive and reactive taxa are distributed along the rank abundance curves.

### Terrestrial influx and aquatic legacy shape reactive and unreactive fractions of bacterioplankton communities

The high connectivity and unidirectional flow within aquatic networks have often been neglected, and the effect of upstream selection history and dispersal among aquatic water bodies as well as from the surrounding terrestrial habitats has rarely been studied together at a whole watershed scale. Based on DNA observations, previous studies that linked terrestrial to aquatic ecosystems have converged to report large contributions of terrestrially-derived taxa within aquatic microbial assemblages [12], especially in systems with short residence times and higher connectivity to the surrounding terrestrial milieu [12–14]. Based solely on the analysis of DNA, we similarly observed a high prevalence of terrestrially-derived taxa along the entire aquatic continuum, but also a clear divergence in community structure between terrestrially-influenced (soil, soilwaters, groundwater) and larger aquatic water bodies (river, reservoirs) along this continuum. Streams, headwater ponds, tributaries and small lakes represented intermediate states between the two endpoint community structures. Notably, community structure of lakes was extremely heterogeneous and possibly reflected variations in the combination of network position [66] and residence time [25]. Clustering of reservoirs with riverine lakes and some larger lakes may indicate that residence time is likely a strong driver of community structure [10, 12, 67].

DNA alone, however, only provides a partial view of the underlying assembly processes as it has been unclear whether the observed strong terrestrial signature in aquatic systems [12] represents passive transport or active selection. After combining DNA-RNA-patterns, we observed that this strong terrestrial imprint is not limited to the unreactive fraction but was even more pronounced in the reactive taxa that we identified along the whole continuum. These terrestrially-derived taxa are likely a mixture of true terrestrial taxa and aquatic taxa that were once dispersed into and subsequently persisted in soils (i.e. seeds) [11, 12]. The only aquatic habitat where the terrestrially-derived taxa did not overwhelmingly dominate the reactive pool was the estuary, where local estuarine taxa became more relevant. Although most reactive taxa could be retraced to soils and soilwaters in all habitats, there were nevertheless taxa that were locally recruited along the aquatic continuum that contributed to the reactive portion, and some taxa first appearing in streams, rivers and reservoirs became reactive downstream, including in the estuary. The comparably small but still relevant contribution of these potentially aquatic taxa to the reactive portion became more evident during our rarefaction test (details not shown, SM4). We observed a larger proportion of stream and upriver taxa (10-25%) in the reactive fraction with lower rarefaction thresholds, indicating that many of the terrestrial taxa are indeed very rare and drop out of the analysis with low rarefaction thresholds. Remarkably, recruitment into the reactive fraction in each habitat occurred across the rank abundance curve, indicating that rare taxa may be highly responsive to the environment and therefore contribute to local ecosystem processes [38, 65, 68]. Overall, the vast majority of taxa that showed local increases in abundance along the aquatic continuum mostly remained unreactive, indicating that mass effects are not only limited to terrestrially-derived taxa but apply similarly to other taxa first detected in aquatic habitats that are carried along the network as a historical imprint [30]. These other taxa may not necessarily be strictly aquatic but could be taxa recruited from other source habitats that were not sampled within this study such as aeolian dispersed, wetlands, biofilms, and/or host-associated microorganisms. These taxa collectively form the historical imprint, which grows as the water traverses through various habitats along the flow path, and hence at any time within the network a fraction of the bulk community embodies previous migration and selection that has happened in upstream habitats.

### Spatial and seasonal shifts in dominant assembly processes

**Fig. 6:**
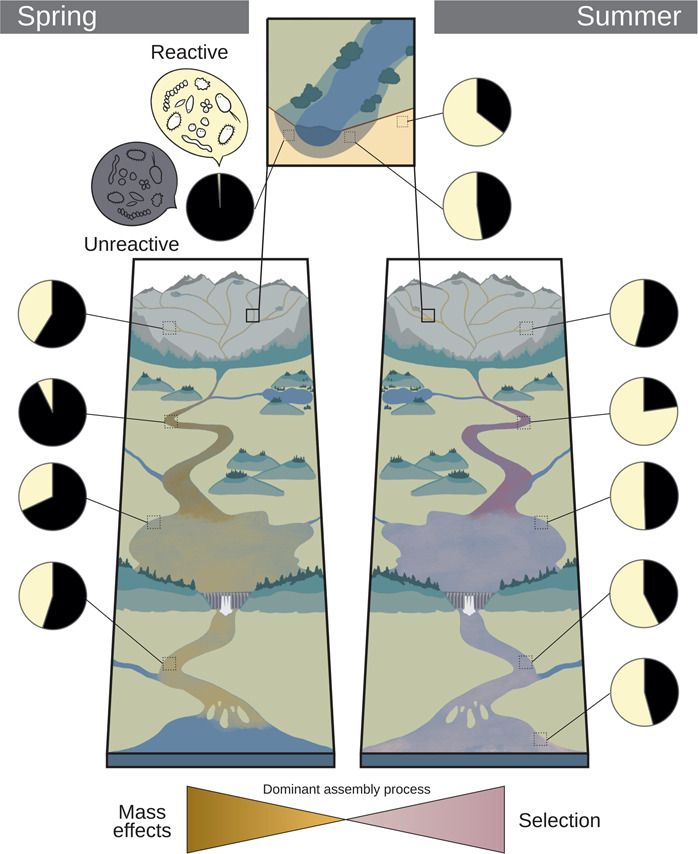
Conceptual figure of seasonally dominant assembly processes along the terrestrial-aquatic continuum. Pie charts visualize the proportion of bacterial OTUs (%) that were identified as reactive and unreactive, respectively (Fig. S11).

DNA- and RNA-based community structures were generally coherent, showing both seasonal as well as spatial gradients in almost a mirroring pattern within the PCoA, which has been observed previously [25, 64], however, their degree of similarity varied greatly in both time and space. As observed in our incidence and abundance-based dissimilarity comparison (Fig. 4), there is a very strong seasonal pattern and a clear spatial structure along the continuum in how DNA and RNA community structures relate to each other. Together with the pattern in reactive versus unreactive taxa, we observed a clear shift in the overall dominant assembly process at the whole network scale between seasons (Fig. 6). Dominance of mass effects in spring and increases in species selection in summer/autumn were likely driven by seasonality in hydrology (e.g., seasonal shifts in discharge (Figs. S12–13) and reservoir residence time (Table S2)) [69], temperature and other environmental factors [70]. Where within the network species selection was most prominent, however, also differed between seasons, as evidenced in the network patterns of dissimilarity between DNA and RNA community structure and in the distribution of reactive and unreactive taxa (Fig. 6). In particular, we observed that reservoirs and subsequent downstream habitats were sites of more intense selection in spring, whereas upstream riverine sites became selection hotspots in summer, albeit selection continued to occur in downstream habitats (Fig. 6). Increasing selective pressure along a riverine residence time gradient has been hypothesized before [34], however, we have shown that the location of strongest selection shifts depending on the hydrological conditions. As such, high flow conditions push selection hotspots downstream, while low flow pulls selection upstream within the network. This seasonally moving window of selection hotspots is likely driven by a balance between the time a water parcel travels along the network and the time a taxon needs to react and grow, which is itself related to temperature and other environmental factors [71].

The only exception from this strong seasonal and shifting spatial pattern were small order streams, which are largely influenced by inputs from the surrounding catchment, as has been shown before [12–14, 18]. Yet, streams were also found to be sites of strong selection independent of season, with consistently high proportions of reactive taxa relative to the connected soilwater communities and relatively large divergence between DNA-RNA-based community structure. Higher growth rates of stream bacterioplankton have been observed previously compared to higher residence time habitats [72], and low order streams have been shown to be sites of intense processing of terrestrially-derived dissolved organic carbon [73]. The shallow environment with a plethora of fresh dissolved organic matter, likely provides unique niches that favor growth and selection of taxa that are washed in from the soil. Stream biofilms may help explain the relatively high selection despite low residence time as they are known to adapt their structures depending on the force of flow [74] and create microhabitats of enhanced microbial growth and biogeochemical processing [75]. It is outside the scope of this study to evaluate biofilms as an additional source for bacterioplankton communities [76], however, biofilms may similarly recruit terrestrial taxa [77]. Our results suggest that the selection processes within these streams likely operates mostly by reshuffling terrestrially-derived taxa rather than selectively recruiting freshwater taxa, as we have observed an overwhelming proportion of terrestrially-derived taxa within the reactive fraction. This stream-filtered, terrestrially-derived community is what becomes the core of the aquatic historical imprint that will be transported throughout the network, representing the basis of dispersal and mass effect to the downstream habitats. This historical imprint is also a source of recruitment along the continuum, thus shaping the whole network scale community all the way into the estuary.

Together, our results suggest a framework wherein low order streams are sites of both intense mass effects and selection across seasons, while further hotspots of selection downstream are modulated by seasonality. As such, high flow conditions only allow for selection hotspots to occur once the network enters longer residence time habitats (i.e. larger reservoirs, lakes) and selection continues to prevail in subsequent downstream habitats such as the river. In contrast, low flow scenarios, which often correspond to higher temperatures and other environmental conditions that favor growth (except under winter low flow conditions, not covered in this study), allow selection hotspots to occur earlier (i.e., upstream river) as travel time decreases. This framework suggests that mass effects are present at all times—regardless of high or low flow—within the network, resulting from both influx of bacteria from the surrounding terrestrial soils and a fluvial carry-over of aquatic bacteria that encompasses the legacy of upstream assembly processes. It is rather the degree and location of selection that can vary significantly in magnitude within the network depending on seasonality. The resulting recruitment of taxa occurs across the rank abundance curve, contradicting the common assumption that only the most abundant bacteria are reactive to the environment and contribute to ecosystem processes. Given the large role that the historical imprint plays in shaping microbial communities within the network, it is not surprising that the various attempts to link microbial processes to the bulk community composition often yield indecisive results [78]. In conclusion, our study highlights the importance of conceptually and empirically considering the potential downstream effect of upstream habitats and the seasonality of these influences when examining the assembly of aquatic microbial communities and the ecological underpinnings of the rank abundance structures observed in aquatic habitats.

## Supplementary information

Supplementary information

## Data Availability

The raw 16 S rRNA gene sequences, both DNA and cDNA are available at the public NCBI Sequence Read Archive (SRA) as part of the BioProject PRJNA693020. The code is available on Github (https://github.com/CarBBAS/Paper_Stadler-delGiorgio_ISMEJ_2021) and both code and processed microbial data were separately archived on Zenodo. [79, 80].
