# Supplementary material for: Terrestrial connectivity, upstream aquatic history and seasonality shape bacterial community assembly within a large boreal aquatic network
Source: ISME J. 2021 Nov 2;16(4):937–47. doi: 10.1038/s41396-021-01146-y (PMC8941091; doi:10.1038/s41396-021-01146-y)
Supplement: Supplementary file 1 — Supplementary information [file 41396_2021_1146_MOESM1_ESM.pdf]

Supplementary information:

Terrestrial connectivity, upstream aquatic history and seasonality shape bacterial community assembly within a large boreal aquatic network

Stadler, Masumi & del Giorgio, Paul A.

Contents

List of Figures 2

Supplementary material and methods 4

SM1: Catchment characteristics . . . 4

SM2: Sample processing and sequencing . . . 5

SM3: Bioinformatic analysis . . . 6

SM4: Effects of rarefaction . . . 7

SM5: Data exploration and statistical analyses . . . 9

SM6: Simulation of theoretical communities to understand underlying patterns of Δ-distances . . . 11

SM7: Abundance classification . . . 14

SM8: Software details . . . 15

Additional supplementary figures 15

Suppplementary Tables 20

References 21

The following document contains the supplementary material for the publication “Terrestrial connectivity, upstream aquatic history and seasonality shape bacterial community assembly within a large boreal aquatic network”. The corresponding R code for the supplementary material can be found on the publication’s Github repository: https://github.com/CarBBAS/Paper_Stadler-delGiorgio_ISMEJ_2021.

24 Pages, 13 Figures, 2 Tables

List of Figures

S1 **Landscape within the Romaine catchment.** (a) Northern area with shrubs and moss-lichen, (b) Mountainous section close to Reservoir 3, (c) Lower coastal plain with peatland areas, (d) Example of a sampled stream. 5

S2 **Comparing the effect of rarefaction on α diversity patterns.** Three different α diversity indices were investigated: Shannon-Wiener index (H’), Simpson’s index (λ) and Pielou’s evenness (*J*). CSS = cumulative sum scaling. Rarefied datasets with the applied minimum library size threshold are indicated as ’Lib[threshold]’. 8

S3 **Distance along all PCoA axes equal pair-wise dissimilarity.** As a proof of concept, the computed distance (e.g. m_{BC}) extracts a proportion of the individual pair-wise dissimilarities, summation of the distances across all PCoA dimensions equals the initial pair-wise Bray-Curtis dissimilarities, which is the input matrix into the PCoA. Thus, computing the pair-wise distance among a sub-selection of axes of the PCoA captures a proportion of the overall pair-wise dissimilarity that is explained by the variation and drivers of the selected axes. 10

S4 Schematic representation of n-distance calculation 11

S5 **Simulated Δ-distances.** Points represent the arithmetic mean of 9 independent simulations of SAD and replacement categorical combinations. Error bars indicate the standard deviation from the arithmetic mean. 12

S6 **Linear regressions of Δ-distances and computed metrics.** Points are arithmetic means for simulations (a-b) and individual samples for empirical data (c-d) (simulation: n = 16, empirical data: n = 193). Assumptions of normality and homogeneity were checked for each linear regression. Lines and grey areas represent regression slope and confidence intervals, respectively. Regression formulae and statistics are given within the individual plots. 13

S7 **Classification of abundance groups.** Schematic representation of the used approach to classify abundance groups based on derivative approximation of the log-transformed rank abundance curve by habitat type, where a) represents the original rank abundance curve and b) the log-transformed equivalent to derive points of maximum and minimum acceleration. The blue point represents the first minimum of the second derivative, red points are the first and second maxima of the second derivative. Pink, green and blue ranges visualise abundant, medium and rare classifications, respectively. 14

S8 **Taxonomic composition of habitat types.** Given are averages of phyla found across habitat types. Upper panel shows average library sizes across habitat types. Error bars indicate the standard deviation from the mean. 15

S9 **No clear seasonal clustering within terrestrial samples.** PCoA analysis with square rooted Bray-Curtis dissimilarity on Hellinger transformed community matrix with only terrestrial samples (n = 156, 11 047 OTUs). Habitat types are distinguished by colour, seasons are indicated by shapes and nucleic acid type are visualised by different line colour. 16

S10 **Habitat type and seasonal separation of RNA.** PCoA analysis with square rooted Bray-Curtis dissimilarity on Hellinger transformed community matrix of all RNA samples (n = 201, 7 549 OTUs). Habitat types are distinguished by colour and seasons are indicated by shapes. . 17

S11 **Unreactive and reactive fractions of OTUs.** Proportion of bacterial OTUs (%) within each habitat type and season that can be attributed to the unreactive (RNA = 0 and < Median) and reactive (> Median) fraction. 18

S12 **Modelled and measured discharge across the watershed.** Discharge was continuously measured at point locations at various stream orders by Hydro-Québec. A model was created to model the discharge of all streams and rivers within the watershed using mean monthly air temperatures (weather station at Havre-Saint-Pierre airport), monthly difference in snow melt (retrieved from Brown and Brasnett (2010)) and flow accumulation (derived via GIS). This model is part of a separate manuscript in preparation, hence, the script and data are not available on Github. 19

S13 **Seasonal differences in discharge across the watershed.** Given are predicted discharges of all lotic systems within the watershed for the years 2015-2017. Spring, Summer and Autumn correspond to the months June, August and October, during which the field campaigns were carried out. Lines represent polynomial linear regressions. These results are part of a separate manuscript in preparation, hence, the script and data are not available on Github. 20

Supplementary material and methods

SM1: Catchment characteristics

The river springs between the Atlantic and Saint Lawrence watersheds (52°52'20"N 63°36'55"W; elevation: 702 masl), and consequently flows through a series of lakes (hereafter riverine lakes) including the biggest lake in the catchment – Lake Brûlé (*A*: 127.11 km^2, elevation: 470 masl). The river mainly flows towards the South with a maximum distance from the northern headwaters to the river mouth expanding to approximately 475.1 km.

The catchment was glaciated 7 000 – 10 000 years ago and left mostly a till blanket and veneer as surficial material. It is mainly dominated by acid rocks (e.g. granodiorite, granite, quart diorite) with granitized sedimentary and volcanic rock, and has isolated patches of permafrost (0-10%)(Natural Resources Canada). The soil is composed of roughly 61.4% sand, 31.9% silt, 6.7% clay and stores approximately 140.4 t ha^{-1} of organic carbon (in top 5 cm; given are catchment averages) (Lehner and Grill (2013), Hengl et al. (2014)).

The northern part of the catchment is characterised by a flat open black spruce (*Picea mariana*)-lichen forest with shrubs and moss-lichen (Fig. S1a). As one follows the river downstream, the relief changes drastically to a steep mountainous stretch that forms sections of canyons (Fig. S1b). The river looses 330 m of elevation from the mountainous section until it makes a sharp turn to the west into the lower coastal plain. The coastal plain is characterised by peatland areas with swamps and shallow waters that are completely permafrost free (Fig. S1c). There are two larger tributaries in the coastal plain that flow through the lakes Puyjalon (*A*: 13.10 km^2) and Allard (*A*: 19.24 km^2). Nearly half of the catchment is covered by coniferous forests (*P.mariana*-moss), with mixed forests being rather minor (11%) and deciduous stands with white birch (*Betula papyrifera*) and trembling aspen (*Populus tremuloides*) are even more rare (2%)(l'environnement and Canadian Environmental Assessment Agency (2009)).

The Romaine river was dammed during the sampling period, forming a reservoir cascade complex with 4 reservoirs by 2020 after the sampling period. The reservoirs Romaine 2 (RO2, *A*: 81.15 km^2, mean depth: 61 m), Romaine 1 (RO1, *A*: 13.22 km^2, mean depth: 22 m) and Romaine 3 (RO3, *A*: 35.18 km^2, mean depth: 66 m) were flooded in the years 2014 (winter), 2015 (winter), and 2017 (spring), respectively.

A weather station located in the lower coastal plain (50° 16'55.000" N, 63° 36'41.000" W, Havre-Saint-Pierre Airport, Natural Resources Canada) recorded an annual precipitation of 810.77 $\pm$ 35.25 mm and 1.18 $\pm$ 0.73 °C, -32.63 $\pm$ 1.36 °C, and 25.8 $\pm$ 0.66 °C for mean, minimum and maximum temperature over the sampled years.

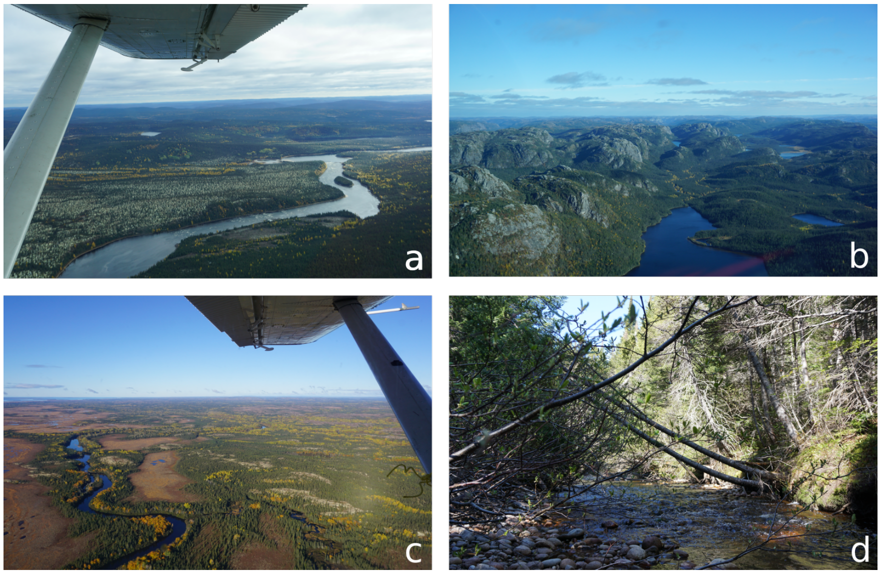

Figure S1: Landscape within the Romaine catchment. (a) Northern area with shrubs and moss-lichen, (b) Mountainous section close to Reservoir 3, (c) Lower coastal plain with peatland areas, (d) Example of a sampled stream.

SM2: Sample processing and sequencing

Surface water samples were directly collected into a pre-rinsed carboy bottle at a depth of 0.5 m, close to the shore for stream samples and diverse locations within the river and reservoirs. Surface soil samples were collected by mixing three randomly selected cores (30 cm) that were taken in proximity of installed piezometers to sample soilwater. The upper 5 cm including surface vegetation were removed before the soil was transferred into a sterile plastic bag. Three piezometers were randomly installed in proximity (30-100 cm) to a sampled stream with an average depth of 50 $\pm$ 20 cm. However, if the piezometers were installed too close to the stream main channel, hyporheic water was sampled instead. Piezometers were emptied 3 times (1-2 h) with a peristaltic pump before sample water was collected. The water from the piezometers was pooled for each site. Groundwater was directly collected from constructed wells with submersible pumps. Lake sediment samples were collected with sediment cores (1-2 m depth), and the upper 10 cm were collected and mixed for subsequent processing. All samples were stored in cooler boxes until return to the laboratory (maximum duration 7h), and were subsequently stored at 4 ℃ upon arrival at the laboratory until further processing on the same day of sampling. A minimum of 25 mL and 250 mL of soil-/hyporheic-water and surface water, respectively, were filtered through 0.22 µm polycarbonate membrane filters (Merck Millipore, Darmstadt, Germany). Homogenized soil and sediment samples were transferred to aliquots of 0.25 g. After filtration, samples for RNA extraction were submerged in RNAlater and LifeGuard Soil Preservation solution (QIAGEN, Hilden, Germany) for water and humic samples (soil, soilwater, hyporheic water), respectively. To

allow stabilisation in the buffer, samples were left at 4 ℃ overnight and were subsequently stored frozen. All DNA and RNA samples were frozen at -20 ℃ at the field station and further stored at -80 ℃ at the university laboratory until extraction.

For extractions, PowerWater and PowerSoil DNA and RNA extraction kits (MoBio, Carlsbad, CA, USA) were used to extract water and soil/soil-/hyporheic-water/sediment samples, respectively. In 2017, the equivalent DNeasy and RNeasy PowerWater Kits (QIAGEN, Hilden, Germany) were used for DNA and RNA samples, respectively, due to discontinuation of the MoBio kit series.

Prior to cDNA reverse transcription, RNA extracts were checked for DNA contamination with a negative PCR test. Subsequently, cDNA was synthesised with a high capacity cDNA Reverse Transcription Kit (Applied Biosystems, Foster City, CA, USA). Successful DNA extraction and cDNA synthesis was evaluated via PCR amplification of the 515F-806R primers (IDT Technologies, Coralville, IA, USA) and DNA concentration was measured with a NanoDrop 2000c (Thermo Fisher Scientific Inc., Waltham, MA, USA).

SM3: Bioinformatic analysis

Primers were removed from 16S rRNA DNA and cDNA (hereafter RNA) data sets using the software *cutadapt* (Version 1.18, Martin (2013)), which allows for the removal of the primer sequence and its variants in their true and complement orientations. Additionally, all reads shorter than 125 nucleotides were removed as they cannot achieve a minimum overlap necessary for paired-end merging in downstream processing.

To identify amplicon sequence variants (ASVs), 16S rRNA amplicon reads were analysed through the DADA2 (Divisive Amplicon Denoising Algorithm 2) pipeline (Version 1.14.1, Callahan, McMurdie, and Holmes (2017)) on R Version 3.6.3 R Core Team (2020)). Read qualities were evaluated for each sequencing plate separately and read length was trimmed according to their quality scores. Samples were pooled by plate, season and sequencing depth for learning the error rates. DADA2 runs on a sample by sample basis, and thus removes observed singletons by sample to avoid inclusion of false-positive sequencing errors. To retain more rare taxa within a sampling campaign (year-season combinations) along the continuum, samples were ‘pseudo’-pooled for the *dada()* step. This step enables the removal of singletons by pool but retains singletons within a sample. Paired-ends were merged after successful inference of amplicon variants. Chimeras were removed (*removeBimeraDenovo()*) and, finally, taxonomy was assigned with the *DECIPHER* package (Version 2.14.0, Wright (2016)) implementing the increased accuracy IDTAXA algorithm Murali, Bhargava, and Wright (2018) and the provided trained classifier of the GTDB database (Release 95, Parks et al. (2018)). Only ASVs that were classified as Bacteria and not as Mitochondria or Chloroplast were evaluated in this study. Several

ASVs were found to be highly abundant only in RNA. To account for slight differences that may have emerged between DNA and RNA ASVs and also to merge potential differences among 16S rRNA copies within a single genome, ASVs were merged into OTUs by a 99% similarity threshold Větrovský and Baldrian (2013) with the *DECIPHER* package (*AlignSeqs(), DistanceMatrix(), IdClusters()*, Wright (2016)). The sequence of the most abundant ASV within a OTU cluster was kept as a reference sequence if it was classified as at least at the domain level as “Bacteria”. If the most abundant ASV within a cluster did not have any taxonomic classification, the ASV that had a taxonomic classification was chosen to represent the OTU cluster.

SM4: Effects of rarefaction

Cumulative sum scaling (CSS) results were compared with results achieved with various rarefaction thresholds. There were no substantial differences in the results between CSS and various rarefaction thresholds on α diversity estimates, which is believed to be most susceptible to library size differences (Fig. S2).

We further re-run all analyses in the manuscript with the examined rarefaction thresholds. Both PCoAs showed negligible differences while the patterns in Sørensen and Bray-Curtis distances showed variation across rarefaction thresholds. Depending on the rarefaction threshold used, we observed different patterns in when incidence and when abundance-based distances were greater than the other, which was likely introduced by the random sampling procedure imposed by rarefaction affecting both incidences as well as abundances. As the threshold becomes higher (e.g. 10000), all patterns in the absolute numbers of Sørensen and Bray-Curtis distances approach our originally observed patterns with CSS. We mainly use the Δ-distances to interpret shifts in mass effects and selection, and while the absolute numbers in Δ-distances changed, the patterns across habitat types and seasons remained fairly consistent across rarefaction thresholds with the highest rarefaction threshold resembling the original results the most. Hence, these results showed that the absolute numbers in Δ-distances do not hold meaning per se, it is the spatio-temporal relative change that gives our analysis meaning. Rarefaction does change the DNA-RNA distances themselves likely due to the loss of rare taxa and making abundance differences among OTUs smaller, however, the final interpretations remain the same.

Finally, we explored the effects of rarefaction on how reactive and unreactive OTUs were identified. Across all rarefaction thresholds, we could observe a clear differentiation between OTUs that were classified within each habitat as ‘unreactive’. Firstly, due to their absence in any RNA and secondly, by the absence in a DNA to RNA contribution relationship. OTUs that were categorized by these two ‘unreactive’ categories were clearly different from those that were named ‘reactive’ due to the presence of a linear relationship in their DNA and RNA contribution. Within each rarefaction threshold, unreactive OTUs were characterized by diverse origins,

and reactive taxa were mainly dominated by soil and soilwater taxa. We could also observe that with lower rarefaction thresholds (e.g. 1470), more taxa from ‘aquatic’ habitats seemed to be reactive (i.e. taxa first detected in streams and upriver), however, as the rarefaction threshold increases, their proportion reduces (e.g. 10000). These results point to the fact that rare taxa are important contributors to our observed patterns, and hence we strongly believe that rarefaction does rather bias our observations by removing taxa essential to natural processes in microbial assembly (e.g. seed banks, Lennon and Jones (2011)). Rarefaction does not affect our observation that there were taxa that have a decoupled DNA-RNA relationship (below potential activity threshold), and those that do (above potential activity threshold). Consequently, CSS results were used for our manuscript.

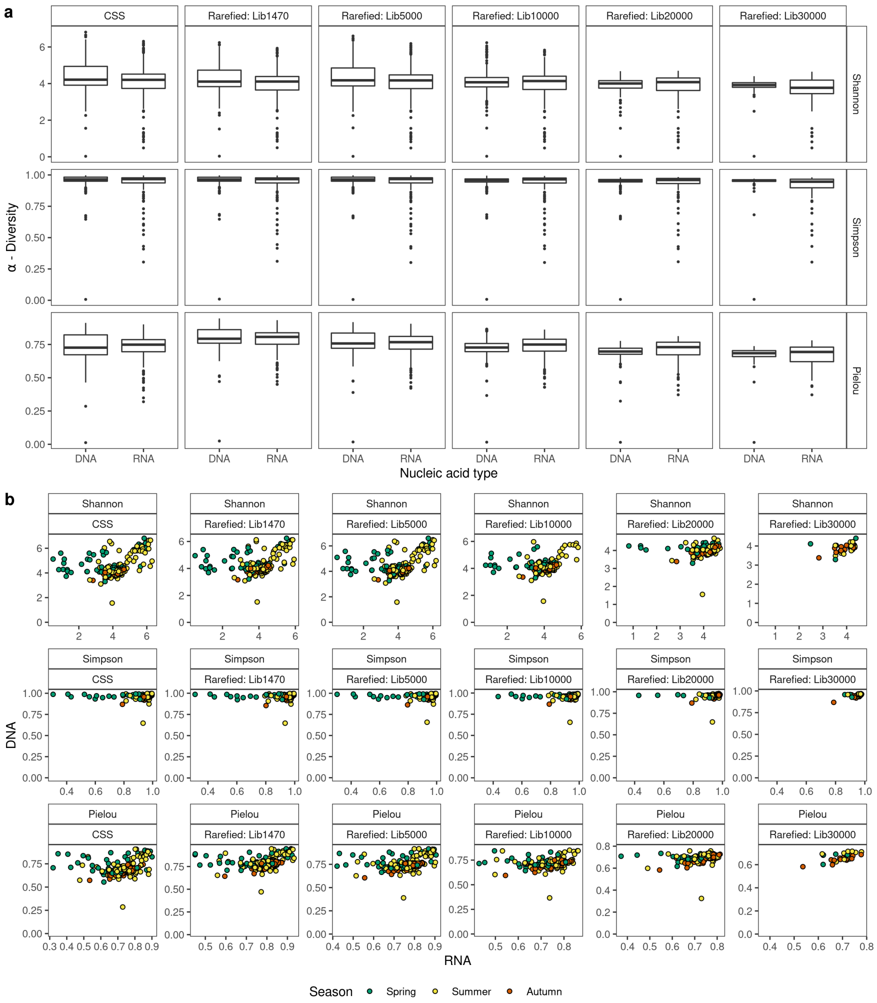

Figure S2: Comparing the effect of rarefaction on α diversity patterns. Three different α diversity indices were investigated: Shannon-Wiener index (H’), Simpson’s index (λ) and Pielou’s evenness (J). CSS = cumulative sum scaling. Rarefied datasets with the applied minimum library size threshold are indicated as ’Lib[threshold]’.

SM5: Data exploration and statistical analyses

To explore differences in microbial community composition across habitat types and seasons, a Principal Coordinates Analysis (PCoA) was conducted with Bray-Curtis dissimilarities (D_{BC}) (Bray and Curtis (1957), Legendre and Legendre (1998)) based on all DNA samples with the function *pcoa* in the *ape* package (Paradis and Schliep (2018)). The community matrix was Hellinger transformed to resolve a horse-shoe effect (Legendre and Gallagher (2001)). To correct any negative eigenvalues problematic for PERMANOVA analysis, the D_{BC} matrix was square-root transformed to Euclidean distance (Legendre and Legendre (1998), Borcard, Gillet, and Legendre (2011)). To evaluate statistical differences in habitat type and season a PERMANOVA was computed with 9999 permutations with the *adonis* function. A PERMANOVA cannot distinguish among-group from within-group variation if data dispersion is variable among groups (Anderson and Walsh (2013)), therefore, an analysis of multivariate homogeneity was computed with *betadisper*. Using *permutest*, we finally tested whether dispersion differs between groups.

Secondly, to evaluate whether sampled RNA-based assemblages were different from the DNA-based assemblages, we performed a second PCoA (D_{BC} with square-root transformation) with both DNA and RNA samples. Again, statistically different groups were investigated with a PERMANOVA (9999 permutations), where habitat type, season and nucleic acid type (DNA vs. RNA) formed the groups. The same framework explained above to check for dispersions was applied. To quantify how different DNA-/RNA-based assemblages of the same sample are, the Bray-Curtis distance (m_{BC}) of each DNA-RNA sample pair within the PCoA ordination space was computed across *n*-dimensional space (Tabak (2004)):

$$m(p,q) = \sqrt{(\mid p_1 - q_1 \mid)^2 + (\mid p_2 - q_2 \mid)^2 + \cdots + (\mid p_n - q_n \mid)^2}$$

where p and q represent DNA and RNA site scores, respectively, of each sample and n is the used maximum number of dimensions. We focused on the first axes that cumulatively explain 75% of the variation for each ordination ($n_{75\%}$), similar to (Osterholz et al. (2016)). This approach was implemented as it was evident from the PCoA that essential variation within non-aquatic samples was captured outside the first three axes. The distance across all PCoA axes equals the initial pair-wise dissimilarity on which the PCoA is based on (Fig. S3). As such, the distance across $n_{75\%}$ axes extracts the proportion of the initial pair-wise dissimilarity that is captured by the axes cumulatively explaining 75% of the PCoA.

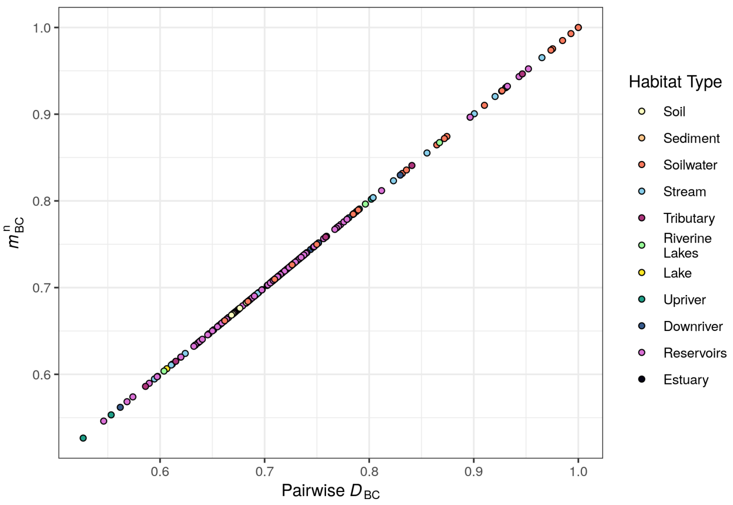

Figure S3: Distance along all PCoA axes equal pair-wise dissimilarity. As a proof of concept, the computed distance (e.g. m_{BC}) extracts a proportion of the individual pair-wise dissimilarities, summation of the distances across all PCoA dimensions equals the initial pair-wise Bray-Curtis dissimilarities, which is the input matrix into the PCoA. Thus, computing the pair-wise distance among a sub-selection of axes of the PCoA captures a proportion of the overall pair-wise dissimilarity that is explained by the variation and drivers of the selected axes.

To gain further insight into the processes shaping assemblage dissimilarities, we computed a PCoA with the Sørensen dissimilarity (D_S), which is the incidence based equivalent of D_{BC} (square-root transformed to achieve Euclidean space) (Legendre and Legendre (1998), Sørensen (1948))(Fig. S4). By comparing incidence and abundance based dissimilarities, we can further distinguish in which samples DNA-/RNA-based assemblages diverge primarily due to different present taxa or their abundances, respectively. We further applied the same framework of calculating the distance among DNA and RNA pairs across $n_{75\%}$ axes resulting the Sørensen-based distance (m_S).

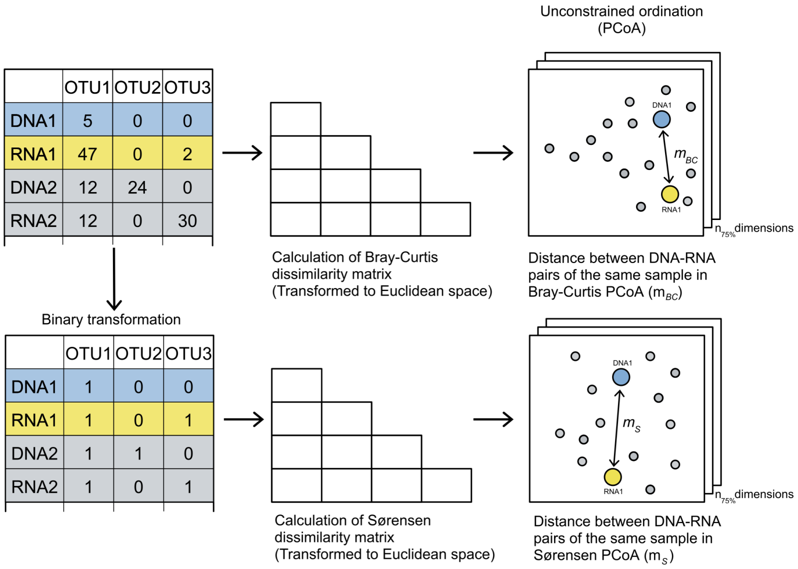

Figure S4: Schematic representation of n-distance calculation

SM6: Simulation of theoretical communities to understand underlying patterns of ∆-distances

In order to support our approach to examine assembly processes using the ∆-distances approach between Sørensen and Bray-Curtis dissimilarity based PCoAs, we simulated different scenarios and calculated the ∆-distances on these theoretical communities.

We used four different species abundance distributions (SADs) of varying evenness (Pielou's J) to mimic the DNA assemblages of different habitat types. We hypothesized that the degree of selection increases with lower evenness (higher ∆-distance), as only a few taxa are selected for and dominate the community. Each SAD was composed of 10000 OTUs (Lennon et al. (2018)), which is close to our overall OTU pool of 16322 OTUs found across the dataset. We used lognormal distributions to create SADs and modified the evenness by changing the scale parameter between 0.9 and 2.7. The higher the parameter is, the steeper and less even the community becomes. To create the DNA assemblages, we randomly sampled 25000 times from each SAD, which was based on our average library size across the dataset (mean: 24687.21).

As a second step, we duplicated the randomly sampled DNA assemblages to retrieve a base community for the corresponding RNA assemblages. To introduce additional mass effects, we implemented a gradient of OTU removal from the created DNA assemblages. The number of OTUs removed from the RNA assemblages were either 1/2, 1/3, 1/6 or 1/9 of all sampled OTUs in a site (hereafter, replacement). We hypothesized that

the higher the replacement, the stronger the mass effect will be (lower Δ-distance). The number of reads that were removed as a result of the random OTU removal were re-sampled from the OTUs that were not present in the DNA assemblage of a particular site, leading to equal library sizes across sites. We further corrected for phantom taxa (RNA > 0, DNA = 0) with DNA = 1, following the analysis of our empirical dataset. Overall, we implemented 4 SAD and 4 replacement treatments that were run 9 times to avoid random sampling biases and compute standard deviations of the resulting metrics (overall n = 288).

Once the OTU matrix with all sites were set-up, we re-ran our analysis by calculating the PCoAs for both Sørensen and Bray-Curtis based dissimilarities, extracted the axes that cumulatively explain 75% of the variation and calculated the Δ-distances. Furthermore, two metrics were calculated for each site: 1) the number of OTUs that do not have any RNA, which were classified as "unreactive" taxa in our study, and 2) the mean read difference between individual OTU's DNA and RNA, which indicates the discrepancy between the DNA and RNA. We hypothesized that higher DNA-RNA discrepancies indicate stronger selection.

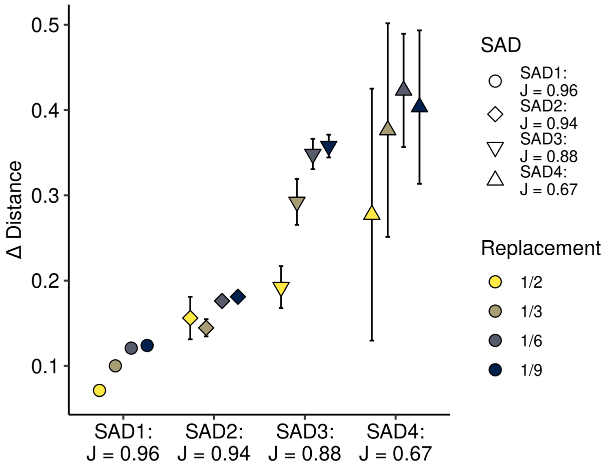

Figure S5: Simulated Δ-distances. Points represent the arithmetic mean of 9 independent simulations of SAD and replacement categorical combinations. Error bars indicate the standard deviation from the arithmetic mean.

Our simulation results indicate that there is indeed a clear trend in our evenness and replacement treatment on Δ-distances with lower Δ-distances observed in even communities (i.e. stronger mass effect) and higher Δ-distances in uneven communities (i.e. stronger selection) (Fig. S5). Additionally, higher replacement values lower the Δ-distances within each SAD treatment indicating stronger mass effects.

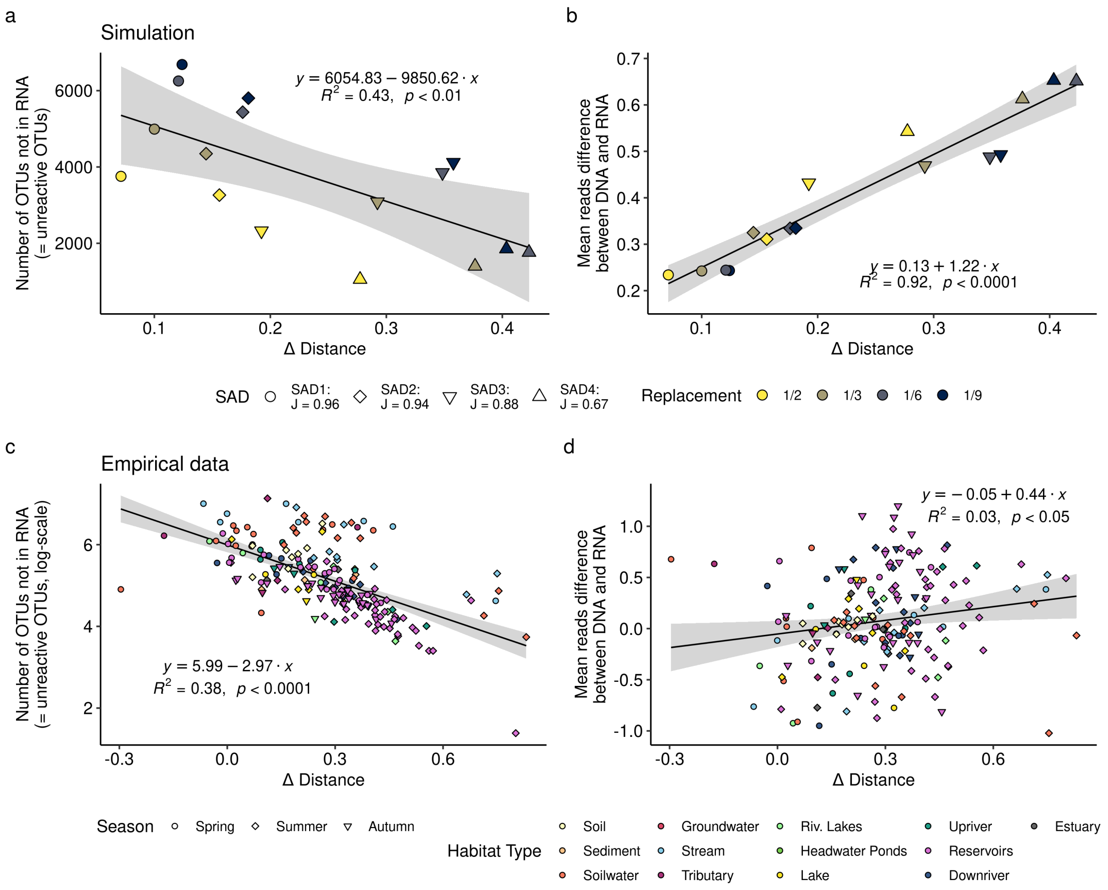

Figure S6: Linear regressions of ∆-distances and computed metrics. Points are arithmetic means for simulations (a-b) and individual samples for empirical data (c-d) (simulation: n = 16, empirical data: n = 193). Assumptions of normality and homogeneity were checked for each linear regression. Lines and grey areas represent regression slope and confidence intervals, respectively. Regression formulae and statistics are given within the individual plots.

We also regressed the two above mentioned metrics with the ∆-distances (Fig. S6), and we found a negative relationship of the number of "unreactive" OTUs with ∆-distances in line with our interpretation of mass effects and its dependency on a higher proportion of unreactive taxa. Furthermore, mean abundance differences were positively related to ∆-distances indicating stronger selection with higher abundance differences between DNA and RNA. Although our empirical relationships (Fig. S6 c-d) are less stronger than the simulation based estimates (Fig. S6 a-b), the overall trends are the same.

We would like to end this simulation section by highlighting that the absolute number of ∆-distances is likely to hold little meaning. Absolute ∆-distance values are not interpretable on their own, it is the comparison across habitats or gradients that create patterns and gives this approach room for interpretation.

SM7: Abundance classification

Traditionally, abundance groups (AGs) such as “abundant” and “rare” have been defined by various relative abundance thresholds ranging from 0.1 to 1 % within the literature. While inconsistencies hinder comparisons among studies, we additionally are working with variance stabilised read numbers, thus traditional thresholds based on relative abundances are not applicable. In order to classify OTUs into AGs, we developed a new framework to classify OTUs into AGs based on the shape of rank abundance curves of each habitat. We initiate the framework by calculating the mean abundance of each OTU by habitat type. Subsequently, for each habitat type a smoothed rank abundance curve was generated with the function *smooth.spline* with the smoothing parameter set to 0.7 in base R (Fig. S7). Ranks that correspond to moments of acceleration along the curve were identified by taking the second derivative of the log(x + 1) transformed abundance curve (Fig. S7c). All OTUs ranked below the second maximum acceleration were defined as rare. OTUs falling above the first maximum acceleration were defined as abundant, while the section in between the two maxima represents moderately abundant OTUs (Fig. S7a). The CSS reads corresponding to the ranks identified for the AGs were extracted for each habitat type separately. Subsequently, the average CSS reads for each abundance threshold was calculated. This approach classified all OTUs with >= 72 CSS reads as abundant, < 72 and >= 10 CSS reads as moderate, and < 10 CSS reads as rare.

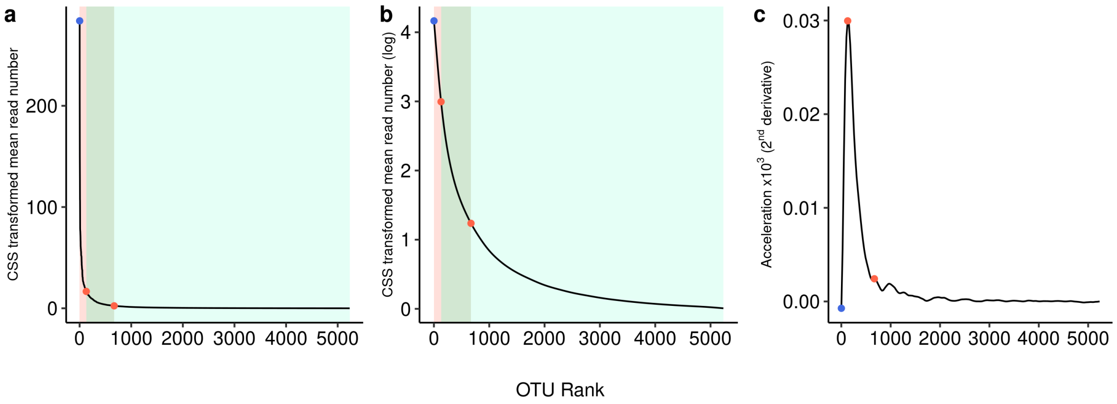

Figure S7: Classification of abundance groups. Schematic representation of the used approach to classify abundance groups based on derivative approximation of the log-transformed rank abundance curve by habitat type, where a) represents the original rank abundance curve and b) the log-transformed equivalent to derive points of maximum and minimum acceleration. The blue point represents the first minimum of the second derivative, red points are the first and second maxima of the second derivative. Pink, green and blue ranges visualise abundant, medium and rare classifications, respectively.

SM8: Software details

The packages *phyloseq*, *tidyverse*, *plyr* and *data.table* were used for data wrangling and transformation (McMurdie and Holmes (2013),Wickham et al. (2019), Wickham (2011), Dowle and Srinivasan (2019)), and *doMC* and *parallel* enabled parallel processing (Revolution Analytics and Weston (2019), R Core Team (2020)). *ggplot2*, *ggpubr*, *ggnewscale* and *cowplot* were used to visualize the results (Wickham (2016), A Kassambara (2020), Campitelli (2020), Wilke (2019)). For statistical analyses, *vegan* and *rstatix* were used (Oksanen et al. (2019), Alboukadel Kassambara (2020)).

Maps were created with QGIS (version 3.12) and a digital elevation model provided by Natural Resources Canada. Watersheds were delineated with ArcMap (version 10.5.1, ESRI Inc., Redland, CA) and the Spatial Analyst Toolbox.

Additional supplementary figures

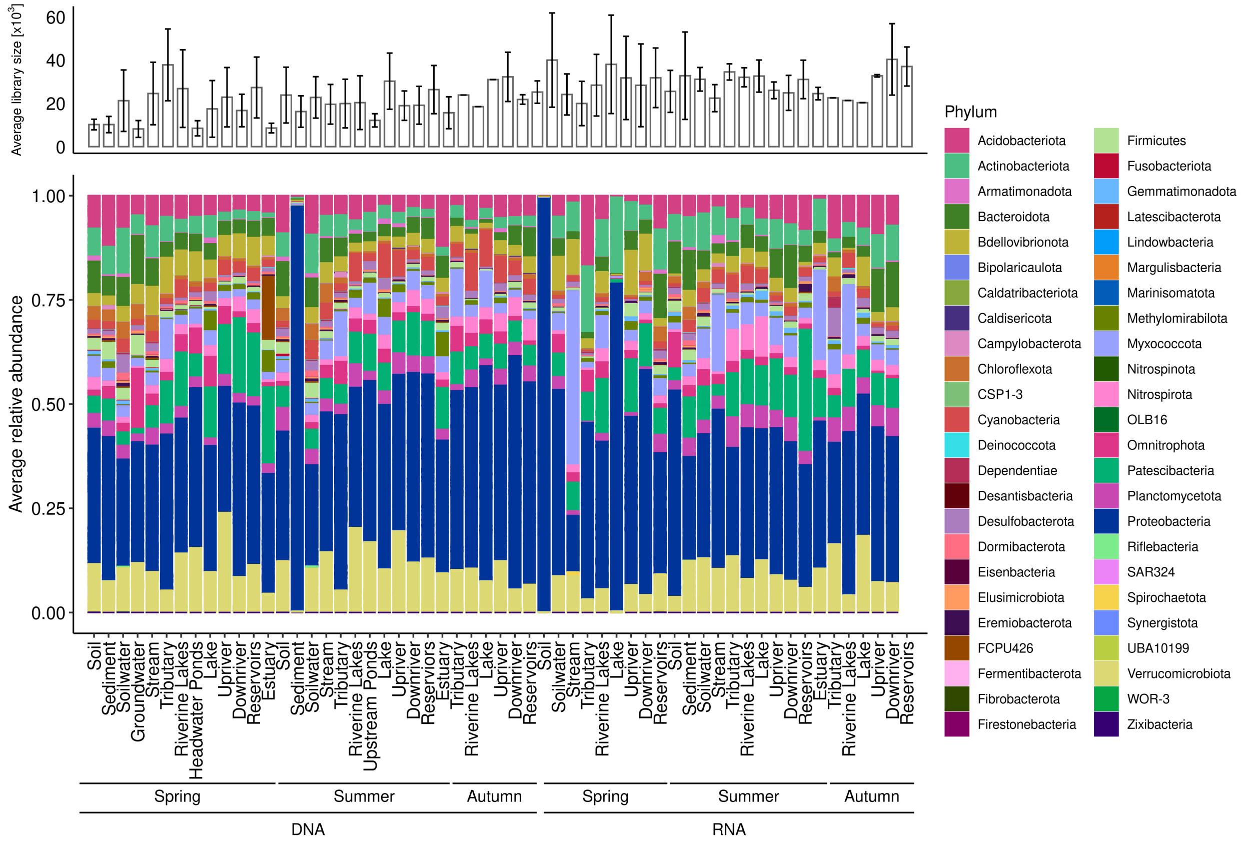

Figure S8: Taxonomic composition of habitat types. Given are averages of phyla found across habitat types. Upper panel shows average library sizes across habitat types. Error bars indicate the standard deviation from the mean.

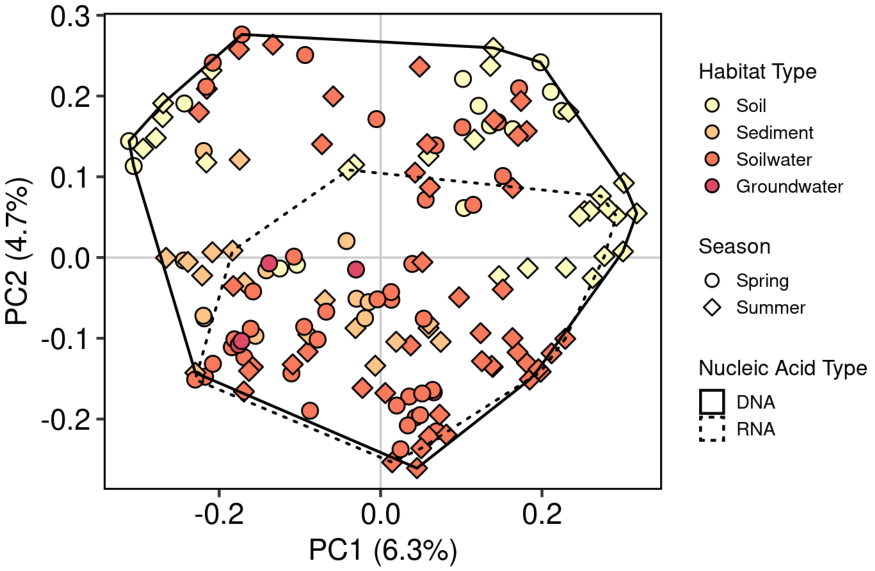

Figure S9: No clear seasonal clustering within terrestrial samples. PCoA analysis with square rooted Bray-Curtis dissimilarity on Hellinger transformed community matrix with only terrestrial samples (n = 156, 11 047 OTUs). Habitat types are distinguished by colour, seasons are indicated by shapes and nucleic acid type are visualised by different line colour.

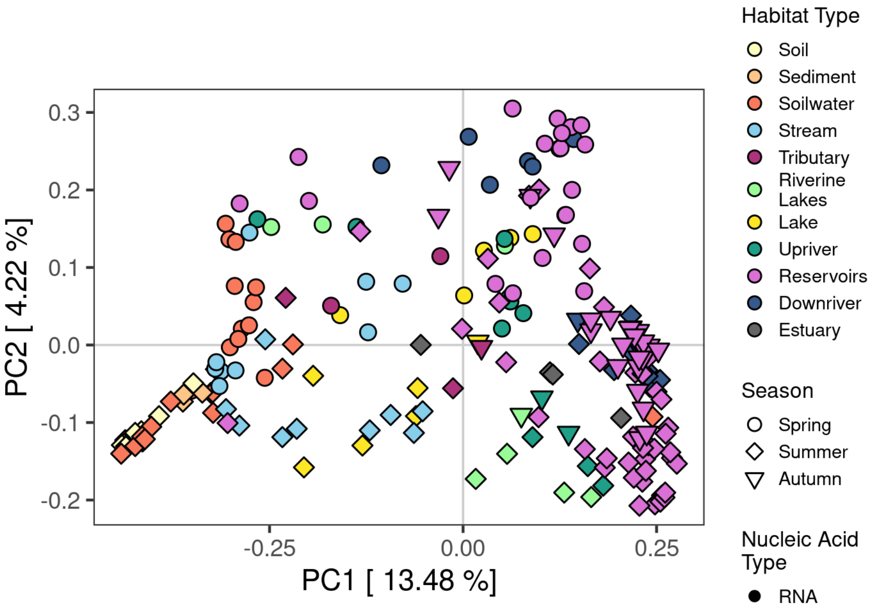

Figure S10: Habitat type and seasonal separation of RNA. PCoA analysis with square rooted Bray-Curtis dissimilarity on Hellinger transformed community matrix of all RNA samples (n = 201, 7 549 OTUs). Habitat types are distinguished by colour and seasons are indicated by shapes.

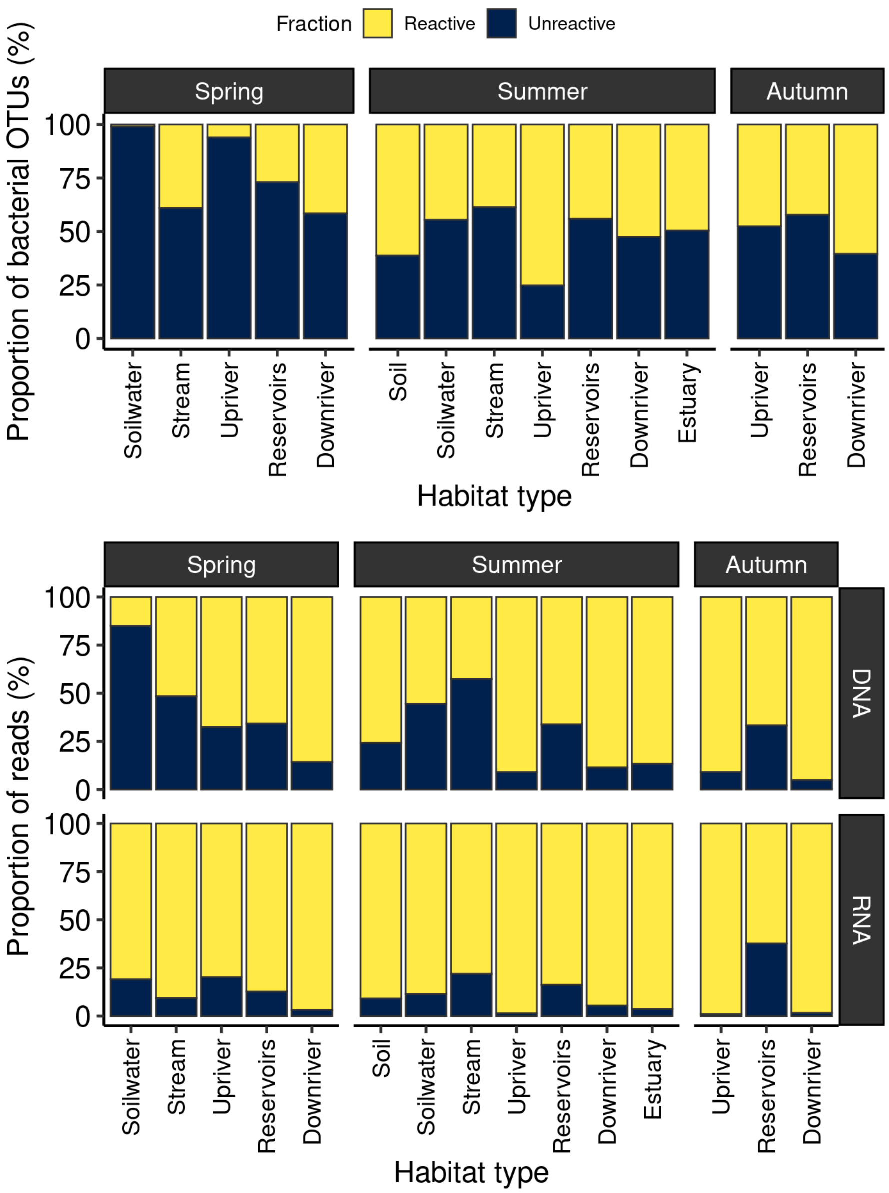

Figure S11: Unreactive and reactive fractions of OTUs. Proportion of bacterial OTUs (%) within each habitat type and season that can be attributed to the unreactive (RNA = 0 and < Median) and reactive (> Median) fraction.

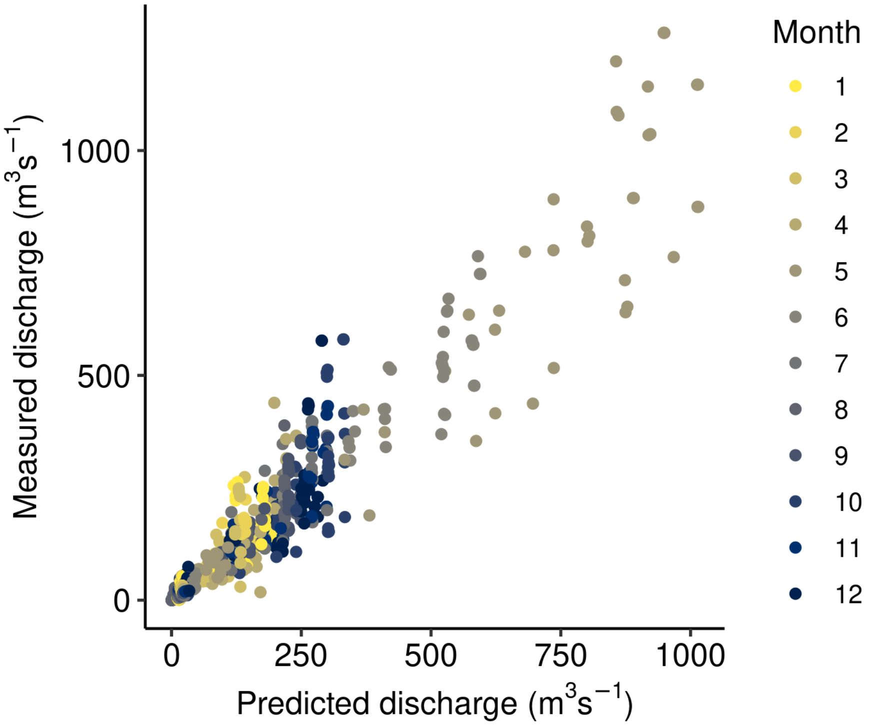

Figure S12: Modelled and measured discharge across the watershed. Discharge was continuously measured at point locations at various stream orders by Hydro-Québec. A model was created to model the discharge of all streams and rivers within the watershed using mean monthly air temperatures (weather station at Havre-Saint-Pierre airport), monthly difference in snow melt (retrieved from Brown and Brasnett (2010)) and flow accumulation (derived via GIS). This model is part of a separate manuscript in preparation, hence, the script and data are not available on Github.

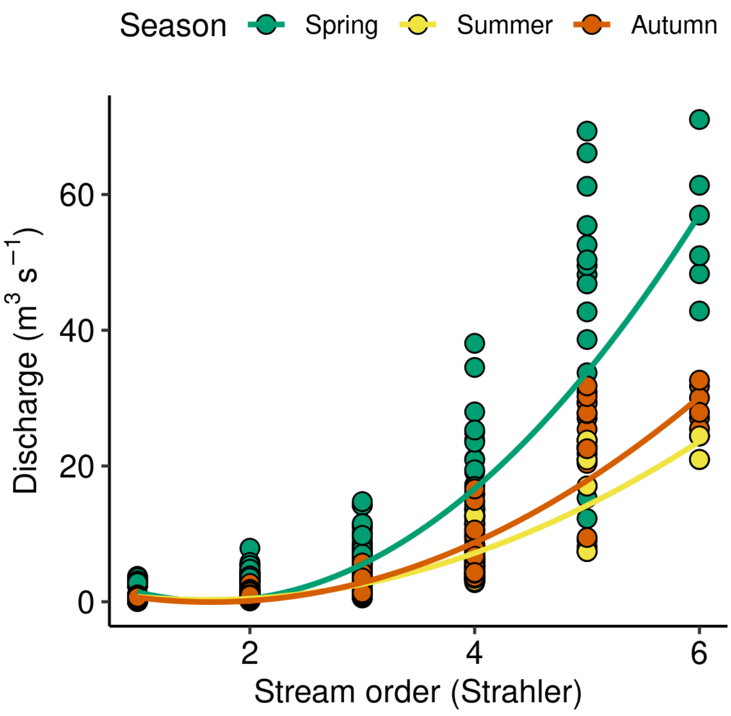

Figure S13: Seasonal differences in discharge across the watershed. Given are predicted discharges of all lotic systems within the watershed for the years 2015-2017. Spring, Summer and Autumn correspond to the months June, August and October, during which the field campaigns were carried out. Lines represent polynomial linear regressions. These results are part of a separate manuscript in preparation, hence, the script and data are not available on Github.

Suppplementary Tables

Table S1: Number of samples per habitat type.

Nucleic Acid Type	Soil	Sediment	Soilwater	Groundwater	Stream	Tributary	Riverine Lakes	Headwater Ponds	Lake	Upriver	Downriver	Reservoirs	Estuary	Sum
DNA	29	22	56	4	33	8	17	10	16	22	50	102	20	389
RNA	8	4	26	0	18	5	8	0	11	12	21	84	4	201

Table S2: Estimated seasonal variation in water residence time (d) across reservoirs. Water residence time is given in days. Water residence time was estimated from reservoir volume and continously measured discharge data at the reservoir outflow by Hydro-Québec. Monthly reservoir volume was estimated from a water level to volume relationship derived by Hydro-Québec. The underlying data are part of a separate manuscript in preparation, hence, the script and data are not available on Github.

Reservoir	Spring	Summer	Autumn
RO1	3.9	6.5	7.4
RO2	90.8	144.2	190.5
RO3	43.8	86.4	115.6

References

Anderson, Marti J., and Daniel C. I. Walsh. 2013. "Anderson and Walsh (2013) PERMANOVA, ANOSIM, and the Mantel test in the face of heterogeneous dispersions- What null hypothesis are you .pdf." *Ecological Monograph* 83 (4): 557–74. https://doi.org/10.1890.

Borcard, Daniel, Francois Gillet, and Pierre Legendre. 2011. *Numerical Ecology with R*. Edited by Robert Gentleman, Kurt Hornik, and Giovanni G Parmigiani. New York: Springer. https://doi.org/10.1007/978-1-4419-7976-6.

Bray, J. Roger, and J. T. Curtis. 1957. "An ordination of the upland forest communities of Southern Wisconsin." *Ecological Monographs* 27 (4): 325–49. https://doi.org/10.2307/1942268.

Brown, R. D., and B. Brasnett. 2010. "Canadian Meteorological Centre (CMC) Daily Snow Depth Analysis Data." Boulder, Colorado USA: NASA National Snow; Ice Data Center Distributed Active Archive Center. https://doi.org/10.5067/W9FOYWH0EQZ3.

Callahan, Benjamin J., Paul J. McMurdie, and Susan P. Holmes. 2017. "Exact sequence variants should replace operational taxonomic units in marker-gene data analysis." *The ISME Journal* 11: 2639–43. https://doi.org/10.1038/ismej.2017.119.

Campitelli, Elio. 2020. "ggnewscale: Multiple Fill and Colour Scales in 'ggplot2'." https://cran.r-project.org/pa ckage=ggnewscale.

Dowle, Matt, and Arun Srinivasan. 2019. "data.table: Extension of 'data.frame'." https://cran.r-project.org/pa ckage=data.table.

Hengl, T., J. M. de Jesus, R. A. MacMillan, N. H. Batjes, G. B. Heuvelink, E. Ribeiro, A. Samuel-Rosa, et al.

2014. “SoilGrids1km—global soil information based on automated mapping.” *PLoS ONE* 9 (8): e105992. https://doi.org/10.1371/journal.pone.0105992.

Kassambara, A. 2020. “ggpubr: ’ggplot2’ Based Publication Ready Plots.”

Kassambara, Alboukadel. 2020. “rstatix: Pipe-Friendly Framework for Basic Statistical Tests.” https://cran.r-project.org/package=rstatix.

Legendre, Pierre, and Eugene D. Gallagher. 2001. “Ecologically meaningful transformations for ordination of species data.” *Oecologia* 129 (2): 271–80. https://doi.org/10.1007/s004420100716.

Legendre, Pierre, and Louis Legendre. 1998. *Numerical Ecology*. 2nd ed. Amsterdam: Elsevier.

Lehner, Bernhard, and Günther Grill. 2013. “Global river hydrography and network routing: baseline data and new approaches to study the world’s large river systems.” *Hydrological Processes* 27 (15): 2171–86. www.hydrosheds.org.

Lennon, Jay T, and Stuart E Jones. 2011. “Microbial seed banks: the ecological and evolutionary implications of dormancy.” *Nature Reviews Microbiology* 9: 119–30. https://doi.org/10.1038/nrmicro2504.

Lennon, JT, ME Muscarella, SA Placella, and BK Lehmkuhl. 2018. “How, When, and Where Relic DNA Affects Microbial diversity.” *mBio* 9 (3): e00637–18.

l’environnement, Bureau d’audiences publiques sur, and Canadian Environmental Assessment Agency. 2009. “Romaine Hydroelectric Complex Project: Investigation and Public Hearing Report.” https://iaac-aeic.gc.ca/050/documents/34211/34211E.pdf.

Martin, Marcel. 2013. “Cutadapt removes adapter sequences from high-throughput sequencing reads.” *EMBnet.journal* 17 (1): pp. 10–12. https://doi.org/10.14806/ej.17.1.200.

McMurdie, Paul J., and Susan Holmes. 2013. “phyloseq: An R package for reproducible interactive analysis and graphics of microbiome census data.” *PLoS ONE* 8 (4): e61217.

Murali, Adithya, Aniruddha Bhargava, and Erik S. Wright. 2018. “IDTAXA: A novel approach for accurate taxonomic classification of microbiome sequences.” *Microbiome* 6 (1): 1–14. https://doi.org/10.1186/s40168-018-0521-5.

Oksanen, Jari, F. Guillaume Blanchet, Michael Friendly, Roeland Kindt, Pierre Legendre, Dan McGlinn, Peter R. Minchin, et al. 2019. “vegan: Community Ecology Package.” https://cran.r-project.org/package=vegan.

Osterholz, Helena, Gabriel Singer, Bernd Wemheuer, Rolf Daniel, Meinhard Simon, Jutta Niggemann, and Thorsten Dittmar. 2016. “Deciphering associations between dissolved organic molecules and bacterial

communities in a pelagic marine system." *The ISME Journal* 10: 1717–30. https://doi.org/10.1038/ismej.2015.231.

Paradis, E, and K Schliep. 2018. "ape 5.0: an environment for modern phylogenetics and evolutionary analyses in R." *Bioinformatics* 35 (526-528).

Parks, Donovan H., Maria Chuvochina, David W. Waite, Christian Rinke, Adam Skarshewski, Pierre Alain Chaumeil, and Philip Hugenholtz. 2018. "A standardized bacterial taxonomy based on genome phylogeny substantially revises the tree of life." *Nature Biotechnology* 36 (10): 996. https://doi.org/10.1038/nbt.4229.

R Core Team. 2020. "R: A language and environment for statistical computing." Vienna. https://www.r-project.org/.

Revolution Analytics, and Steve Weston. 2019. "doMC: Foreach Parallel Adaptor for 'parallel'." https://cran.r-project.org/package=doMC.

Sørensen, Thorvald. 1948. "A method of establishing groups of equal amplitude in plant sociology based on similarity of species content, and its application to analysis of the vegetation on Danish commons." *Biologiske Skrifter Kongelige Danske Videnskabernes Selskab* 5: 1–34. https://doi.org/10.1007/BF02852438.

Tabak, John. 2004. "Differential Geometry." In *Geometry: The Language of Space and Form*, 150. New York: Facts on File, Inc.

Větrovský, Tomáš, and Petr Baldrian. 2013. "The Variability of the 16S rRNA Gene in Bacterial Genomes and Its Consequences for Bacterial Community Analyses." *PLoS ONE* 8 (2): 1–10. https://doi.org/10.1371/journal.pone.0057923.

Wickham, H. 2016. "ggplot2: Elegant Graphics for Data Analysis." New York: Springer-Verlag. https://ggplot2.tidyverse.org.

Wickham, Hadley. 2011. "The Split-Apply-Combine Strategy for Data Analysis." *Journal of Statistical Software* 40 (1): 1–29. http://www.jstatsoft.org/v40/i01/.

Wickham, Hadley, Mara Averick, Jennifer Bryan, Winston Chang, Lucy D'Agostino McGowan, Romain François, Garrett Grolemund, et al. 2019. "Welcome to the tidyverse." *Journal of Open Source Software* 4 (43): 1686. https://doi.org/10.21105/joss.01686.

Wilke, Claus O. 2019. "cowplot: Streamlined Plot Theme and Plot Annotations for 'ggplot2'." https://cran.r-project.org/package=cowplot.

Wright, Erik S. 2016. "Using DECIPHER v2.0 to analyze big biological sequence data in R." *The R Journal* 8

(1): 352–59. https://doi.org/V12242009.
